# Customization Methodology for Conformable Grasping Posture of Soft Grippers by Stiffness Patterning

**DOI:** 10.3389/frobt.2020.00114

**Published:** 2020-09-18

**Authors:** Jun-Young Lee, Jaemin Eom, Sung Yol Yu, Kyujin Cho

**Affiliations:** Biorobotis Laboratory, Department of Mechanical Engineering, Seoul National University, Seoul, South Korea

**Keywords:** soft gripper, pneumatic actuator, stiffness patterning, shape conforming, design customization, pre-grasping posture

## Abstract

Soft grippers with soft and flexible materials have been widely researched to improve the functionality of grasping. Although grippers that can grasp various objects with different shapes are important, a large number of industrial applications require a gripper that is targeted for a specified object. In this paper, we propose a design methodology for soft grippers that are customized to grasp single dedicated objects. A customized soft gripper can safely and efficiently grasp a dedicated target object with lowered surface contact forces while maintaining a higher lifting force, compared to its non-customized counterpart. A simplified analytical model and a fabrication method that can rapidly customize and fabricate soft grippers are proposed. Stiffness patterns were implemented onto the constraint layers of pneumatic bending actuators to establish actuated postures with irregular bending curvatures in the longitudinal direction. Soft grippers with customized stiffness patterns yielded higher shape conformability to target objects than non-patterned regular soft grippers. The simplified analytical model represents the pneumatically actuated soft finger as a summation of interactions between its air chambers. Geometric approximations and pseudo-rigid-body modeling theory were employed to build the analytical model. The customized soft grippers were compared with non-patterned soft grippers by measuring their lifting forces and contact forces while they grasped objects. Under the identical actuating pressure, the conformable grasping postures enabled customized soft grippers to have almost three times the lifting force than that of non-patterned soft grippers, while the maximum contact force was reduced to two thirds.

## Introduction

Softness and flexibility of constituting materials allow soft robotic grippers to be adaptive when interacting with objects (Hughes et al., 2016; Shintake et al., 2018). Recent efforts in this domain have paved ways to implement previously unattainable functionalities of robotic grippers. For example, variable stiffness structures (Amend et al., 2012; Cheng et al., 2016; Wei et al., 2016; Al Abeach et al., 2017; Fei et al., 2018) and friction pads (Zhou et al., 2017; Glick et al., 2018) were integrated into soft fingers; novel materials such as edible gelatin (Shintake et al., 2017), self-healing materials (Cheng et al., 2014; Terryn et al., 2015, 2017), and 3D printable materials (MacCurdy et al., 2016; Mutlu et al., 2017; Hu et al., 2018) were used to fabricate soft fingers. Also, some studies developed new designs of air chamber sections by employing modular approaches or exploiting multiple materials (Milana et al., 2018; Zhang et al., 2018; Park et al., 2019).

Soft grippers, especially those driven by pneumatic actuation, have been in the spotlight for their ability to grasp variously shaped objects and even fragile objects with a simple on-and-off control (Rus and Tolley, 2015; Gorissen et al., 2017). Recent efforts regarding pneumatically actuated soft grippers have resulted in soft gripers appearing in the service and logistics scenes, where the grippers are expected to face unconstrained situations.

On the other hand, industrial sites predefine and constrain every component of their production system settings, to achieve maximum efficiency. Therefore, industrial grippers are most likely to repetitively interact with predefined objects. However, most processes that handle flexible and soft objects are yet to be automated because traditional suction cups and grippers are incapable of safely handling them.

To handle such fragile and soft objects, it is critical to exert a lifting force that matches the weight of the target object; also, contact pressures need to be distributed to reduce concentrated contact forces, which can damage the object. Accordingly, there are studies that control the concentrated contact force through sensor feedback (Su et al., 2020) and increase the contact area between the object and the gripper (Shian et al., 2015; Hao et al., 2018). However, most studies rarely consider the effects of both the contact force and area, simultaneously. In addition, the sensory feedback systems require additional control schemes and resources, which may be burdensome to industrial applications.

**Figure 1:**
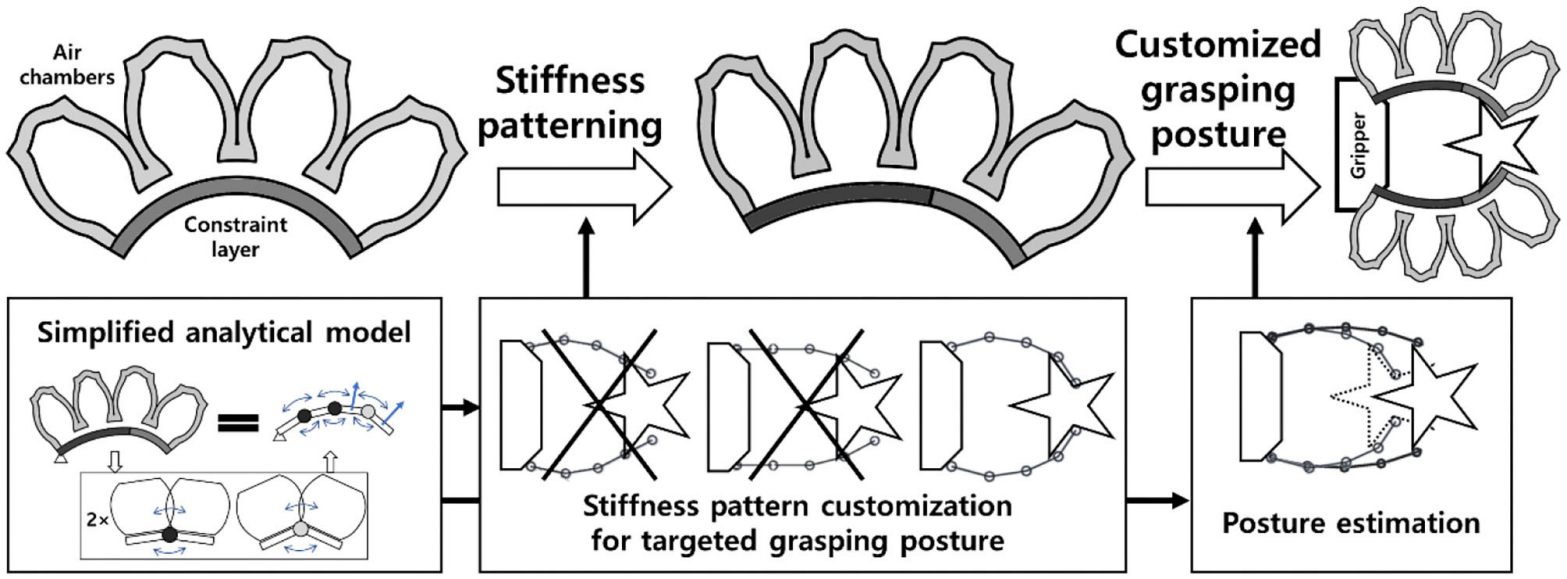
Overview of the stiffness patterning concept and the simplified analytical model for customized grasping postures.

In this paper, we introduce design and fabrication methods to develop customized soft grippers with highly conformable pre-grasping finger postures that outline the shapes of specified target objects (Figure 1) to reduce the contact pressure by increasing the contact area. Implementing grasping postures that match the outlining shapes of target objects has been studied as one way of designing robotic grippers (Shimoga and Goldenberg, 1992; Hurtado and Melkote, 2001; Dollar and Howe, 2006). The adaptiveness of the current soft gripper is manifested when a gripper's structure is deformed upon contacting an object. During this interaction, force is applied to the target object. By implementing conformable grasping postures to soft grippers, grippers can deform to their predefined shapes that outline the target objects without the presence of contact forces. As a result, compared to non-customized soft grippers, the customized soft grippers not only applied the same lifting force with lower actuating pressures but also applied smaller surface contact forces to objects. The lowered actuating pressures and contact forces correspond to efficiency and safety, respectively.

Designing customized soft grippers could be achieved by investigating the bending behavior of a PneuNet-type soft bending actuator, and by engineering the moments generated by the air chamber and constraint layer sections of the soft bending actuator. PneuNet-type actuators have been widely used in the field of soft robotics, and their design could intuitively be segmentized into a series of air chambers. In addition, fabrication of the PneuNet-type actuators is divided into two main parts: the upper air chamber section and the bottom constraint layer section. The constraint layer refers to the section attached to the air chamber section, as shown in Figure 1. Geometric approximations and the pseudo-rigid-body modeling theory were employed to formulate the simplified analytical model. The analytical model describes the interaction between the air chambers and the constraint layer as intersections of moment surfaces. The moment surfaces can be modified and tuned to establish intended conforming grasping postures, by patterning the stiffness of the bottom constraint layer.

The main contributions of this paper are as follows:

- Design methodology for customized soft grippers that are matched to the shape outlines of target objects by patterning stiffness of constraint layers. This approach enables customization without changing the main form factors of the gripper.
- Fabrication methods for customized soft grippers with a modular design approach and a thickness tuning mold approach. The approach enables the manufacturing process of the customized grippers to be cost- and time-efficient.
- A simplified analytical model that estimates and customizes the conforming grasping postures of pneumatically actuated soft bending actuators. The model proposes an insight to analyze and engineer moment surfaces to customize grasping postures. Implementation of the analytical model into the design and fabrication of customized soft grippers with conforming grasping postures.

This report is organized as follows. Section Simplified Analytical Model for Posture Estimation presents a simplified analytical model. In Section Stiffness Patterning of Constraint Layers, discussions about the moment surfaces of soft grippers are presented. Section Fabrication Process of Customized Soft Grippers outlines the fabrication processes of customized soft grippers. Finally, in Section Experimental Results for Customized Soft Gripper, experimental results and comparisons between customized soft grippers and non-patterned soft grippers are presented.

## Simplified Analytical Model for Posture Estimation

**Figure 2:**
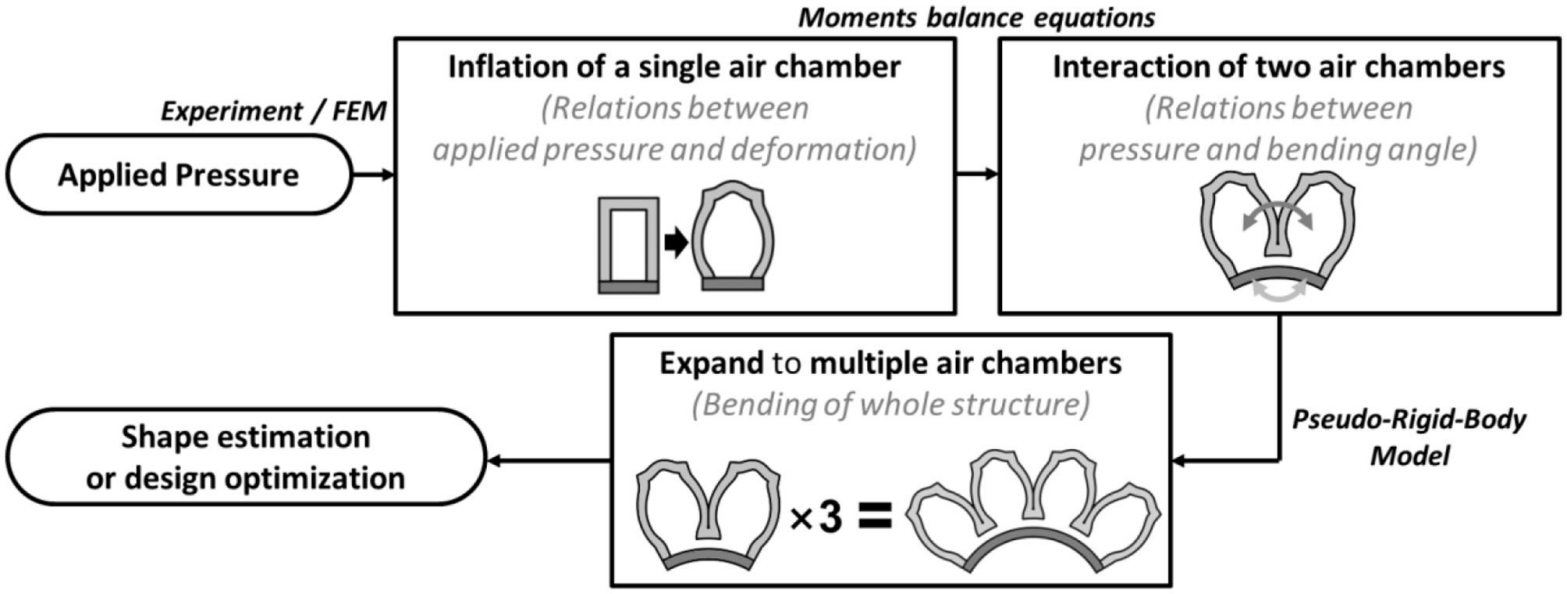
Overview of the concept and the computation process behind the proposed simplified analytical model of soft bending actuators.

Building an analytical model that represents the soft actuator's entire structure is challenging. Our approach is to divide the soft bending actuator into a serial arrangement of interactions between two adjacent air chambers. Then, the chain algorithm for the Pseudo-Rigid-Body (PRB) model (Howell, 2001; Pauly and Midha, 2006a,b) was employed to expand and apply the two-chamber interaction into the whole configuration of the soft bending actuator (Figure 2).

### Model for a Single Interaction Between Two Air Chambers

**Figure 3:**
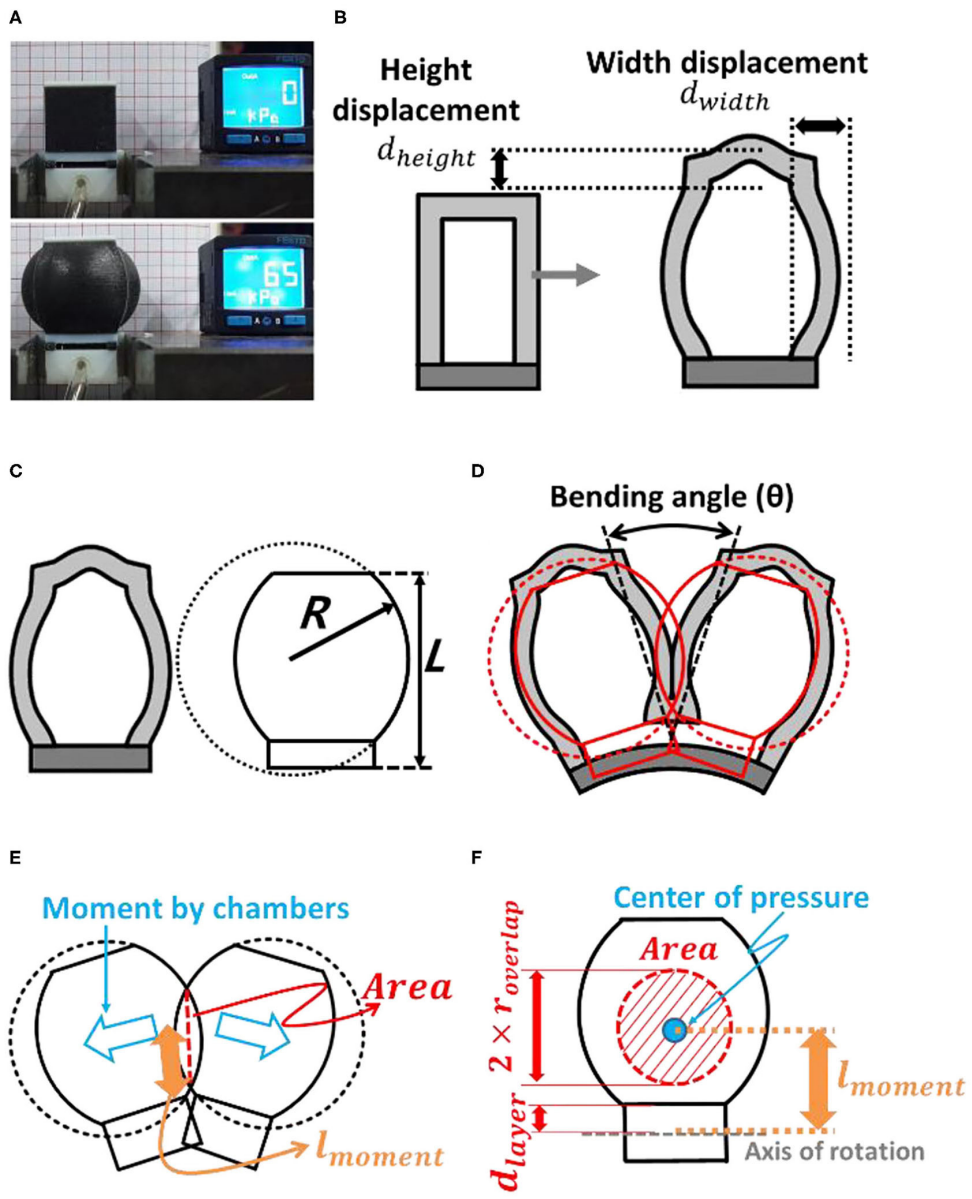
**(A)** Inflation of a modular design single air chamber block. **(B)** During inflation, the heights of the chamber and its side walls were deformed. **(C)** Geometric approximation of the inflated air chamber wall. **(D)** Schematic diagram of the interaction between two air chambers. **(E)** Contact surface and moment arm. **(F)** Interaction of the face.

The first step of the simplified model was to investigate the inflation behaviors of a single air chamber. During inflation, the height and width of the air chamber walls are deformed, as shown in Figure 3. In this paper, the height (*d*_*height*_) and width (*d*_*width*_) displacements due to wall inflation are determined based on the fitting curves obtained via experimental measurements (Supplementary Figures 2, 3, 7), due to the non-linearity of hyper-elastic materials. In future works, it may be possible to investigate the inflation behaviors of a single air chamber with numerical analysis methods.

The bending motion of a soft finger is initiated when two air chambers push each other as they come into contact with each other, after being inflated. In this research, we use chambers with square adjacent facets, which have an approximately circular shape when inflated, as shown in Figure 3. The interaction between the two air chambers may exhibit non-linear characteristics that originate from the material properties and irregular distortion of the inflated chambers. This non-linear distortion of the air chambers was assumed to be negligible, and the model consisted of two circular balloons pushing against each other. Based on this geometric approximation, the radius of the inflated chamber wall (*R*) can be obtained as a function of *d*_*height*_ and *d*_*width*_ as **Equation (2)**. Height of the single air chamber *L* can be described with initial height (*L*_*initial*_) and displacement by inflation (Figure 3B).

(1) $$\begin{array}{l} L=L_{initial}+d_{height} \end{array}$$

(2) $$\begin{array}{l} R=\frac{d_{width}}{2}+\frac{L^{2}}{8\cdot d_{width}} \end{array}$$

The bending moment, *M*_*chamber*_, originated from the pushing force between air chambers, could be described using geometric approximations. The moment generated by the air chambers (*M*_*chamber*_) is described by applied inflating pressure *P* as in **Equation (3)**.

(3) $$\begin{array}{l} M_{chamber}=Area\cdot P\cdot l_{moment} \end{array}$$

(4) $$\begin{array}{l} Area=\pi \cdot {\left(r_{overlap}\right)}^{2} \end{array}$$

(5) $$\begin{array}{l} r_{overlap}=2\cdot \sqrt{R^{2}-{\left(\begin{array}{c} \left(\frac{L}{2}+d_{layer}\right)\cdot \text{sin}\left(\theta /2\right)\\ +\frac{\sqrt{-L^{2}+4\cdot R^{2}}\cdot \text{cos}\left(\theta /2\right)}{2} \end{array}\right)}^{2}} \end{array}$$

(6) $$\begin{array}{l} d_{layer}=\{\begin{array}{c} const.\;=d_{layer,module}\;\left(for\;modular\;design\right)\\ \frac{t_{layer}}{2}\;\left(for\;elastomer\;molding\;design\right) \end{array} \end{array}$$

(7) $$\begin{array}{l} l_{moment}=\frac{\sqrt{\begin{array}{c} 2\cdot L\cdot d_{layer}+2\cdot R^{2}+2\cdot d_{layer}^{2}\\ +\text{cos}\left(\theta \right)\left(L^{2}+2\cdot L\cdot d_{layer}-2\cdot R^{2}+2\cdot d_{layer}^{2}\right)\\ -L\cdot \text{sin}\left(\theta \right)\cdot \sqrt{4\cdot R^{2}-L^{2}}-2\cdot d_{layer}\cdot \text{sin}\left(\theta \right)\cdot \sqrt{4\cdot R^{2}-L^{2}} \end{array}}}{2} \end{array}$$

The overlapping area of the interaction of the two air chambers in Figure 3D was also assumed to have a circular shape. The radius of the overlapped area (*r*_*overlap*_) could be described based on geometric approximations. The moment arm (*l*_*moment*_) could be obtained from the geometric relations derived from the distance between the bottom of the air chamber and the axis of rotation (*d*_*layer*_). The bending of the constraint layer was considered as pure bending. Therefore, the neutral plane of this layer was assumed to be located at its center.

During the bending motion, the constraint layer at the bottom of the structure also generated a moment to return back to the initial state. Based on the Pseudo-Rigid-Body model theory, the constraint layer was assumed to be a non-linear torsional spring. Therefore, the moment generated by the bottom layer (*M*_*layer*_) can be described using the non-linear torsional spring coefficient (*k*_*layer*_) and the bending angle (θ). The non-linear spring coefficient could be obtained from the fitting curve obtained by the three-point bending experiments (Supplementary Figure 3). The moment generated by the constraint layer could be described as a function of the bending angle according to **Equation (8)**.

(8) $$\begin{array}{l} M_{layer}=k_{layer}\left(\theta \right)\times \theta =f\left(\theta \right) \end{array}$$

Finally, the bending angle between two air chambers at a given actuation pressure is computed by solving the moment equivalent equation between *M*_*layer*_ and *M*_*chamber*_. Details regarding the relationship between *M*_*layer*_ and *M*_*chamber*_ will be discussed in chapter 3.

### Expand to Multiple Air Chambers

**Figure 4:**
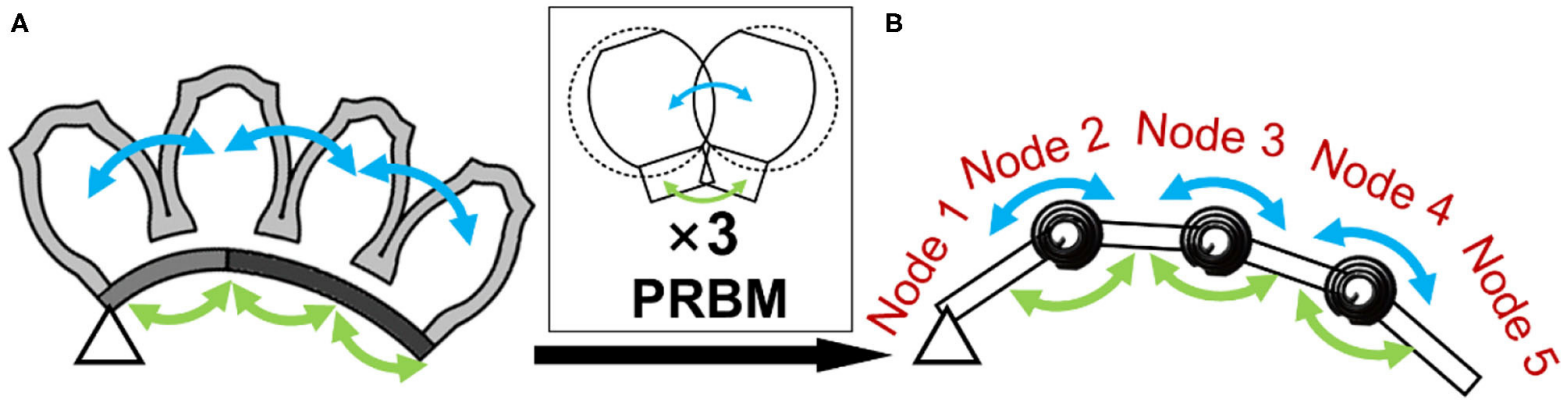
**(A)** Cross-sectional view of a four-air chamber-long soft bending actuator. **(B)** Pseudo-rigid-body model-based approximation of the soft bending actuator. Arrows indicate the moment associated with the air chambers and the layers, respectively.

Based on the Pseudo-Rigid-Body model theory, the entire structure of the soft bending actuator could be considered as a superposition of the interactions between two air chambers that are arranged in the longitudinal direction (Figure 4). Therefore, the bending posture could be represented by the bending angle of each node of the structure. The bending angles could be determined by solving the moment equivalent equations for each node (Algorithm 1). As a result of the simplified analytical model, the posture of the entire structure at any given actuation pressure could be obtained within tens of seconds. For future work, the model could be also applied to other kinds of soft actuators that can be segmentized into a series of interactions between force implying elements (e.g., air chambers) and constraint elements (e.g., constraint layers).

**Algorithm 1:** Estimating actuated posture of soft bending actuator.

**Input**: *p*_*input*_ // Actuation pressure *S* = [*s*_1_, s_2_, …, *s*_*n*_] // Stiffness pattern of the soft gripper with *n* number of nodes **Output:** *G*_*shape*_ = [θ_1_, θ_2_, …, θ_*n*_] // Posture of the soft finger represented by the bending angle of each node 1: **for** *i* := 1 **to** *n* **Do** 2: *d*_*height, i*_ ← FunctionHeight(*p*_*input*_); // Calculate height displacement of a single air chamber at a given actuation pressure 3: *d*_*width, i*_ ← FunctionWidth(*p*_*input*_); // Calculate width displacement of a single air chamber at a given actuation pressure 4: **Find** θ_*i*_ **that** 5: *M*_*chamber, i*_(θ_*i*_, *p*_*input*_, *d*_*height, i*_, *d*_*width, i*_) = *M*_*layer, i*_(θ_*i*_, *s*_*i*_); // Solve the equation about moments generated by the chambers and layer at the *i*-th node 6: **end for** 7: **return** *G*_*shape*_ = [θ_1_, θ_2_, …, θ_*n*_]

## Stiffness Patterning of Constraint Layers

### Analysis of Moment Surfaces and Intersection

**Figure 5:**
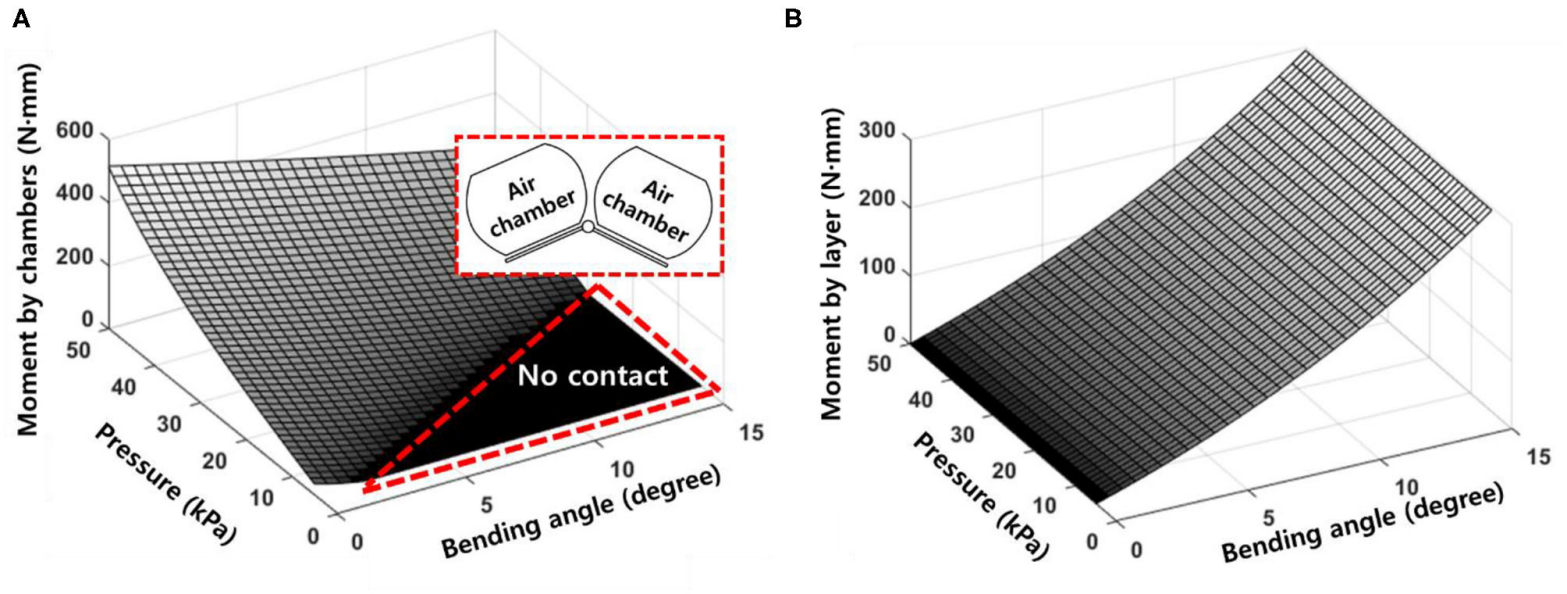
**(A)** Moment surface generated by the air chambers. The no contact area indicates when the air chambers do not contact each other. **(B)** The moment surface generated by the bottom layer. The moment generated by the bottom layer is only related to the bending angle.

The moment generated by the air chambers (*M*_*chamber*_) can be represented by the applied pressure (*p*) and the bending angle (θ), as shown in Figure 5A. The region where the magnitude of the moment is zero represents the instance when the air chambers are inflated but do not contact each other. However, the moment generated by the constraint layer that is plotted in Figure 5B is only related to the bending angle (θ).

**Figure 6:**
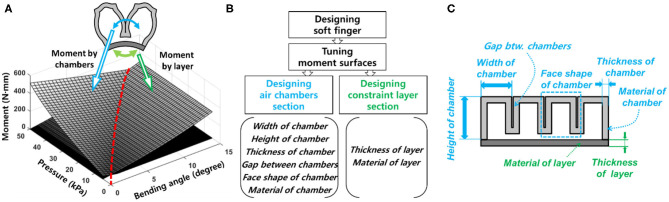
**(A)** The intersection between the moment surfaces (red dashed line) provides the characteristics regarding bending properties of a soft finger. **(B)** Design process for a soft finger. The bending properties and the contact response can be modified by tuning the moment surfaces through designing each section. **(C)** Design parameters for a soft finger.

Figure 6A illustrates the intersection that occurs when the two moment surfaces are plotted together. The intersection characterizes the configuration of the soft bending actuator when there is no contact with the environment. Besides, Figure 6 implies that the shape of the intersection can be modified by tuning the moment surfaces.

### Modifying Moment Surfaces to Design Soft Grippers

Moment surfaces can be tuned through engineering the air chambers and the constraint layer. The air chamber design is affected by the width, cross-sectional shape, thickness of the chamber shell, constituting material, etc. (Figure 6). The design parameters are related to both the air chambers' inflation process and the pushing interactions between the air chambers that generate bending moment. On the other hand, the constraint layer has fewer design parameters than the air chambers. Design parameters for the constraint layers mainly relate to the bending stiffness that resists the bending motion generated by the air chamber section.

Designing the air chambers is relatively difficult than designing the constraint layer section. The air chambers non-linearly deformed and push against each other as they get inflated. However, the constraint layer section only experiences bending motion, but neither inflation nor interaction. Therefore, designing the constraint layer was relatively easy than designing the air chamber section, despite the inherent non-linearity of the material. Furthermore, modifying the air chamber section with different designs may require different molds for fabrication; therefore, changing the design parameters of the constraint layers has several advantages over the air chamber section in terms of manufacturing.

### Stiffness Patterning for Conformable Grasping

By tuning the constraint layer design, the equilibrium point of each node can be modified, which directly affects the overall configuration of the fingers. In other words, the proper arrangement of nodes, each with different stiffness, in the constraint layer is crucial for establishing conformable pre-grasp postures for specified target objects. In this paper, we will call this arrangement the stiffness patterning of the constraint layer. Most conformable stiffness patterns for predefined objects can be found with the analytical model and the genetic algorithm, provided by MATLAB (MathWorks, Inc.) (Algorithm 2). A stiffness pattern that minimizes the mean square error between the shape of the actuated soft gripper and object was selected as the customized pattern design. The outlining shape of the target objects was imported into the algorithm as a fitting curve function and a set coordinates. In this paper, we used three different levels of stiffness patterns: 4, 6, and 8 mm of thickness, as presented in section Model for a Single Interaction between Two Air Chambers of the Supplementary Material. The proposed analytical model enables the customization of stiffness patterns within tens of seconds with a personal computer that has general specifications.

**Algorithm 2:** Stiffness pattern customization based on a target object.

**Input**: *p*_*input*_ // Actuation pressure *O*_*shape*_ // Shape of a target object **Output:** *S* = [*s*_1_, s_2_, …, *s*_*m*_] // Stiffness pattern of a soft gripper with *m* number of nodes to maximize conformability for *O*_*shape*_ while actuated at *p*_*input*_ 1: **Genetic Algorithm:** Find *S*_*i*_ s.t. minimize *error*_*shape*_ 2: *S*_*i*_ ← GetPopulation() // Generate the population for the i-th evolution 3: *G*_*shape,i*_ ← ShapeEstimator(*p*_*input*_, *S*_*i*_) // Get estimated grasping posture of the soft gripper with the stiffness pattern set, *S*_*i*_, at actuation pressure *p*_*input*_ 4: *error*_*shape,i*_ ← MSE(O_shape_, G_shape,i_) // Calculate mean square error between *O*_*shape*_ and *G*_*shape,i*_ 5: **end Genetic Algorithm** 6: **return** *S* = [*s*_1_, s_2_, …, *s*_*m*_]

### Concept of Stiffness Patterning

**Figure 7:**
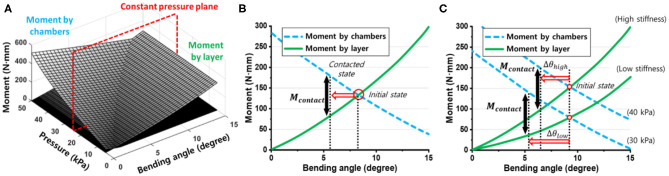
**(A)** Constant pressure plane at a given actuation pressure for a modular design soft finger. **(B)** The moment generated by a contact force (*M*_*contact*_) shifts the equilibrium state from the initial state to the contacted state. **(C)** Comparison between high-stiffness and low-stiffness patterns using modular design soft fingers. When the same contact moment (*M*_*contact*_) was applied to soft fingers with different stiffness patterns, soft fingers with high and low stiffness patterns did not bend at θ_*high*_ and θ_*low*_, respectively.

Stiffness patterning affects the shape of the constant pressure plane of the moment surfaces (Figure 7A). Equilibrium between the moment generated by the air chambers and the constraint layer results in a bending angle that represents the initial steady state of the soft finger without any contact force. However, when the soft finger contacts an object, a moment generated by the contact (*M*_*contact*_) is applied to the finger. The moment due to contact shifts the steady state from the initial state to the contacted state, as shown in Figure 7B.

Without any contact, the soft finger with a high stiffness pattern requires a higher actuation pressure than the soft finger with a low stiffness pattern, to achieve the same amount of bending. As shown in Figure 7C, when the same amount of the contact moment (*M*_*contact*_) is applied to both the high-stiffness and low-stiffness patterned soft fingers, the straightening angles of each stiffness pattern (θ_*high*_, θ_*low*_) are different. The straightening angle of the soft finger with a high stiffness pattern (θ_*high*_) is smaller than that of the soft finger with a low stiffness pattern (θ_*low*_).

There are trade-offs between the low-stiffness and high-stiffness patterns in terms of the bending and straightening characteristics. Table 1 shows a qualitative comparison and the trade-offs between high- and low-stiffness patterns. In the end, it is possible to design actuators that have the same overall appearance by combining different pressures and stiffness. However, because these complementary relationships exist, it is important to properly customize the stiffness pattern according to the specified conditions, such as maximum applicable pressure and the weight of a gripping object.

**Table 1:** Qualitative comparison between low stiffness and high stiffness patterned layers.

**Low-stiffness patterned layer**	**High-stiffness patterned layer**
Large bending angle per applied pressure	Small bending angle per applied pressure
Small energy consumption for required grasping posture	Large energy consumption for required grasping posture
Weak against external distortion	Strong against external distortion
Weak against sagging by object weight	Strong against sagging by object weight
More adaptive to environment	Less adaptive to environment

## Fabrication Process of Customized Soft Grippers

In this chapter, the fabrication process for the customized soft grippers is presented. The aforementioned constraint layer design customization method yields a relatively simple fabrication process for soft grippers.

### Single Mold Fabrication Process for Customized Soft Grippers

**Figure 8:**
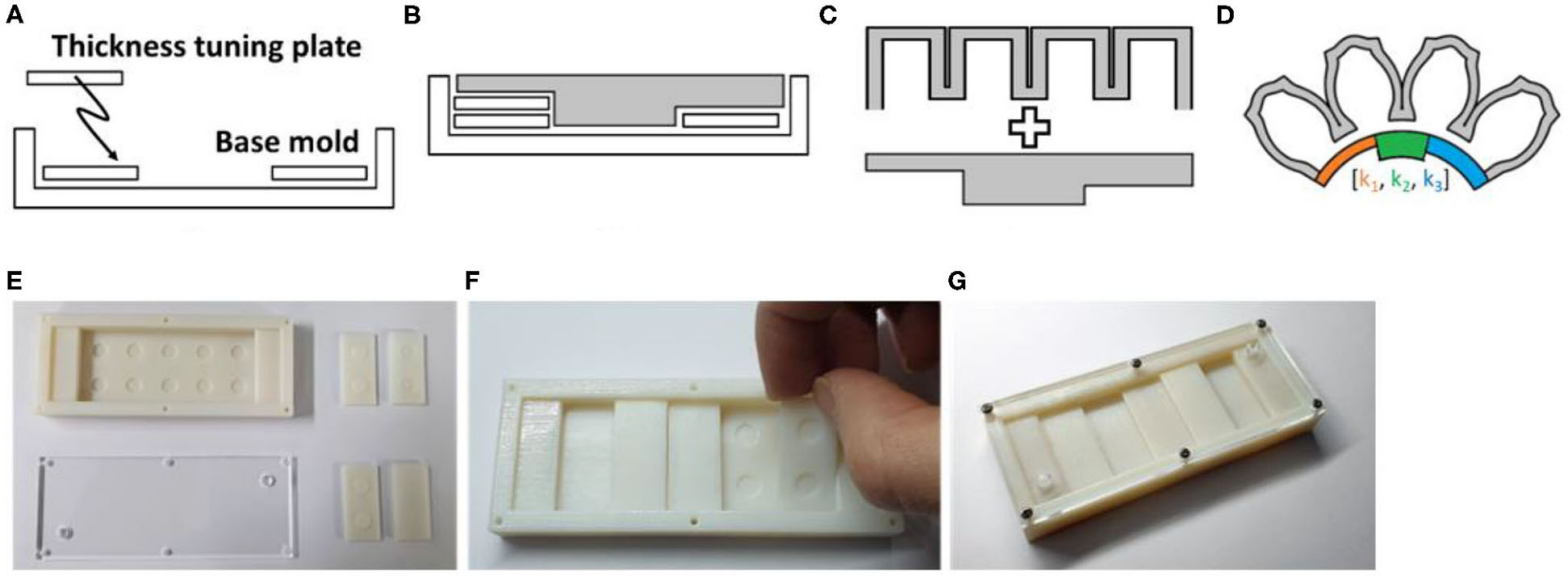
Fabrication process of non-uniform stiffness varying patterned layer. **(A)** The thickness tuning plates are stacked inside the base mold. **(B)** The procured elastomer poured inside the assembled mold. **(C)** The air chamber section and constraint layer are bonded together. **(D)** Soft finger with stiffness patterned layer. **(E)** 3D-printed base mold and thickness tuning plates. **(F)** Thickness tuning plates are stacked inside the base mold. **(G)** Fully assembled constraint layer mold.

Fabricating customized soft grippers may present the presupposition of using customized molds for different designs. However, we present a single mold fabrication process for customized soft grippers. The single mold refers to the fabrication of the air chamber section. As mentioned in section Modifying Moment Surfaces to Design Soft Grippers, modifying the stiffness of the constraint layer, to change the actuated posture, is relatively easy compared to changing the air chamber section's design. Therefore, the stiffness of the constraint layer is modified using a varying stiffness pattern mold, then it is bonded to the air chamber section, which stays constant for different designs. The following outlines the fabrication process of a customized soft gripper: first, the thickness tuning plates are stacked inside the base mold of the constraint layer, according to the desired design; then, a pre-cured elastomer is poured into the assembled mold and cured; finally, the fully cured constraint layer is bonded together with the air chamber section (Figure 8). The resulting soft gripper achieves customized actuated postures with irregular bending curvatures at different pressures compared to their non-patterned counterparts (Supplementary Figure 6).

**Figure 9:**
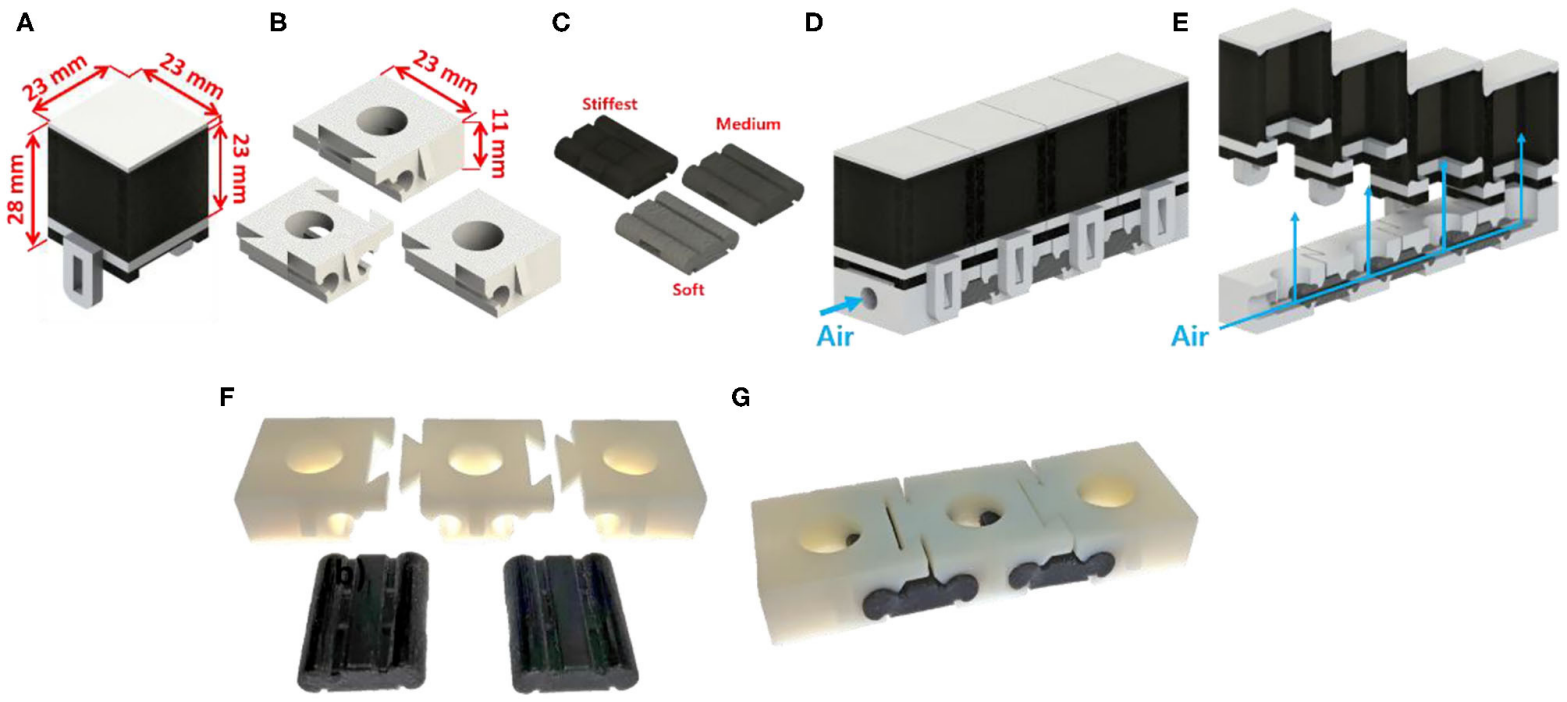
Modular design for customized soft gripper. **(A)** Air chamber block. **(B)** Three kinds of bottom blocks. **(C)** Flexure block. **(D)** Soft finger built by assembling module blocks. **(E)** Cross-section view of the soft finger. Blue arrows indicate actuating pressure. **(F)** Preassembled state of flexure and bottom blocks. **(G)** Assembled bending block.

Also, a stiffness patterning method using modularized blocks is introduced in Figure 9. The idea of using modularized soft robotic blocks was introduced in previous research (Lee et al., 2016, 2018). In this paper, we have implemented stiffness patterning into the previous concept of using modular blocks by using flexure blocks with different stiffnesses (Figure 9C). The advantage of the modular design is that it simplifies the tuning of the stiffness patterns into a process of disassembly and rejoining of the blocks. Details are described in section Expand to Multiple Air Chambers of the Supplementary Material.

## Experimental Results for Customized Soft Gripper

**Figure 10:**
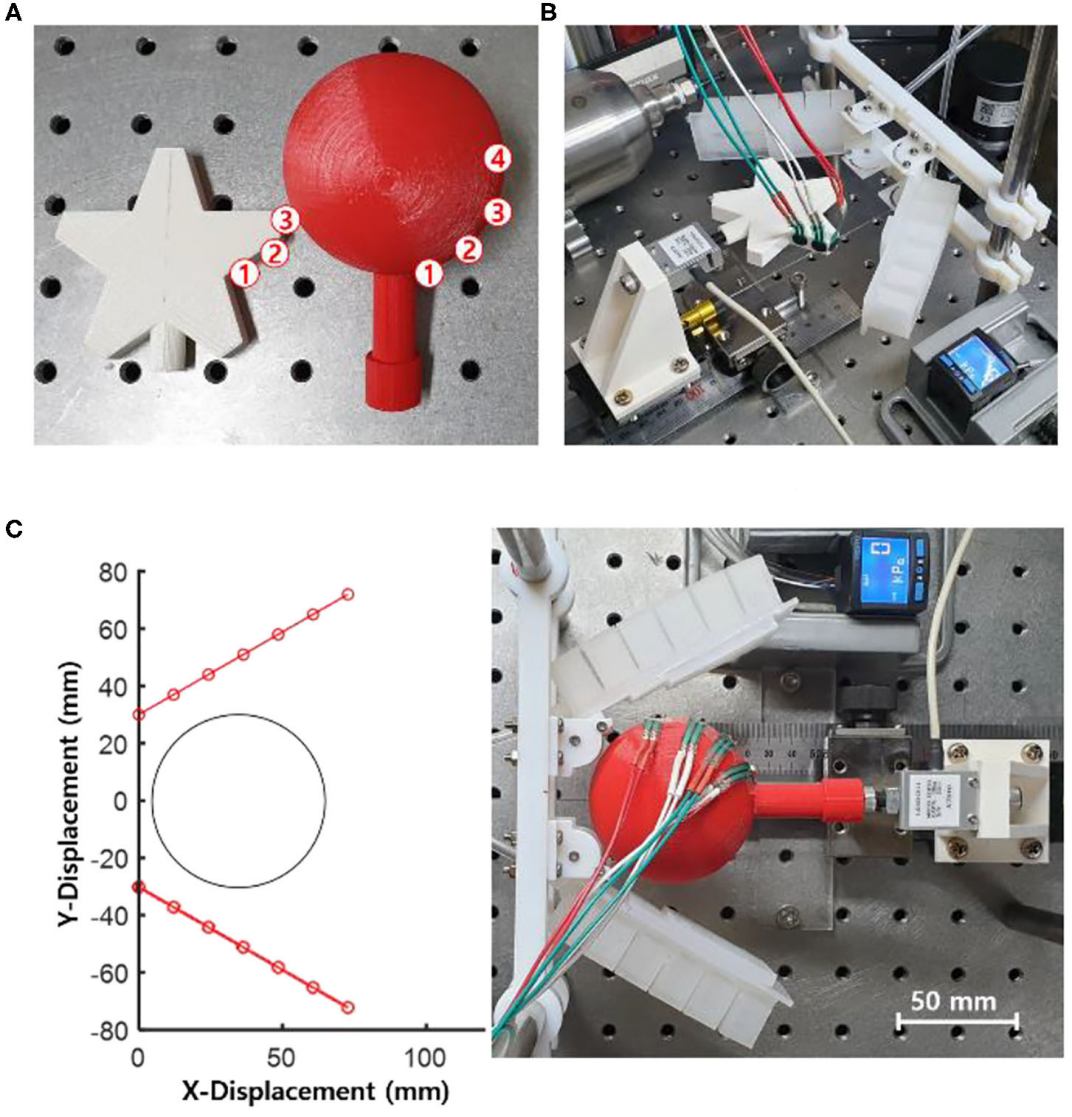
Experimental setup for object gripping and pulling tests. **(A)** Star-shaped and spherical objects were prepared for the pulling tests. FSR sensors were attached to the surfaces of the objects. The circled numbers indicate the locations of the sensors. **(B)** Experimental setup for the star-shaped object. **(C)** View of the experimental setup using the spherical object and the soft gripper with the stiffness patterned soft fingers before actuation.

In this chapter, the experimental results that compare soft grippers with stiffness patterned constraint layers and soft grippers with typical non-patterned homogeneous constraint layers are presented. The experiment consisted of measuring the pulling forces of the soft grippers while grasping target objects. Each gripper had two identical soft fingers mounted in a single plane parallel to the ground (Figure 10). The stiffness patterns were optimized based on the target objects. The actuation pressure was maintained at a constant value throughout the experiment.

### Experimental Setups for Object Grasping and Pulling Tests

Soft grippers with stiffness patterned constraint layer and soft grippers with typical non-patterned homogeneous constraint layers were tested. Both kinds of grippers were fabricated using the same material (Dragon Skin 30, Smooth-On Inc.). In addition, the designs of the air chamber section for both the patterned and non-patterned grippers were identical. The dimensions of the soft fingers were the same as those of the soft fingers presented in Supplementary Material Chapter 2.

Two kinds of objects were selected as target objects. One was a star-shaped object, and the other was a sphere-shaped object (Figure 10). These shapes were chosen because they are very well-known structures, each lying on either end of the extremes: one with no angles and the other with multiple concave acute angles. Both objects were 3D printed using ABS material and fused filament fabrication method. The star-shaped object had a maximum width of 65 mm whereas the sphere-shaped object had a diameter of 60 mm. The objects were connected to a load cell (333FDX, Ktoyo Co. Ltd.) which was mounted onto a linear guide. Force-sensitive resistor sensors (FSR 400, Interlink Electronics, Inc.) were placed on the surface of the objects. The grippers and the objects were designed to be bisymmetric about their center lines. Therefore, the sensors were only attached to a single side of the objects.

Each experiment measured the pulling force and the contact forces between an object and a gripper while the gripper was actuated, and the object was pulled in the outward direction. A load cell and FSR sensors were used to obtain the pulling force and the contact force values, respectively. A draw-wire displacement sensor (CWP-S500R, CALTSensoR) was attached to the same mount where the load cell was positioned. The soft grippers were fabricated based on optimized stiffness pattern designs for each target object.

### Customization of Stiffness Pattern for a Given Target Object

The stiffness pattern designs of the constraint layer were optimized for each target-grasping object at preselected actuation pressures. Customization of stiffness patterns was completed within tens of seconds with a personal computer that has general specifications. This rapid speed of calculation was enabled by the proposed analytical model.

The experimental setup in a two-dimensional plane space is shown in Figure 10C. The soft fingers of the gripper were placed 60 mm apart. The initial angles of the fingers were rotated 30° in the outward direction from the centerline of the grippers. The centers of the spherical and the star-shaped objects were placed 35 and 55 mm apart from the base of the soft grippers, respectively.

**Figure 11:**
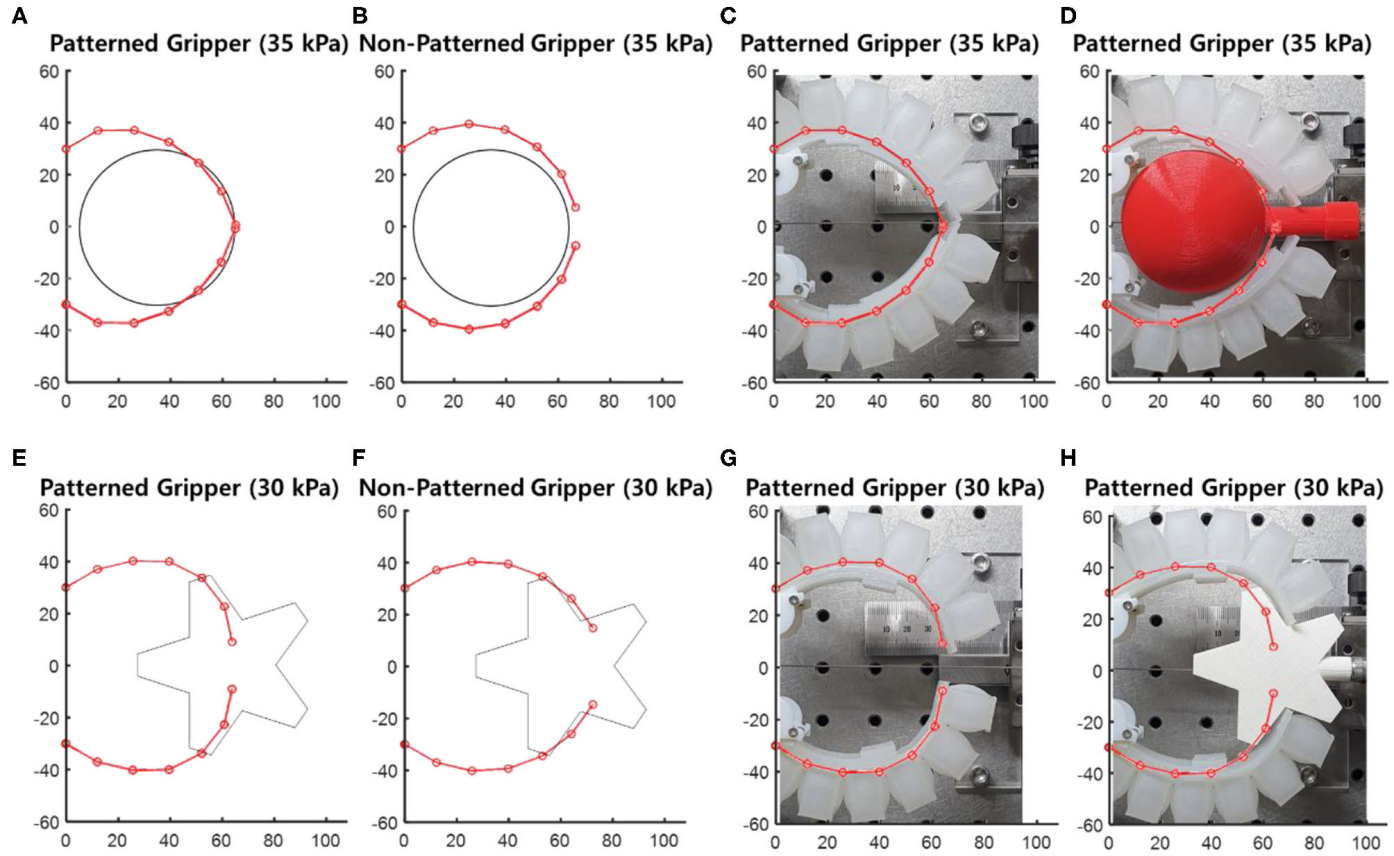
Four soft grippers and spherical object with 60 mm diameter, and star-shaped object. The center of the objects was located at 35, 60 mm from the base of the grippers, respectively. **(A,E)** Customized soft gripper. **(B,F)** Non-patterned soft gripper. **(C,G)** Comparison between experimental and simulated results without the object. **(D,H)** Comparison between the experimental and simulated results with the object.

Figure 11 shows the soft grippers with customized stiffness patterns and those without stiffness patterns. The actuated pressure was selected to be 35 kPa because it resulted in better conformability compared to other pressure values. The customized stiffness pattern at this actuating pressure was 4, 6, 8, 8, and 8 mm, from the proximal node to the distal node. However, the constraint layer of the non-patterned soft gripper was also selected to maximize the conformability to the target object. Based on the simulated results, the soft gripper with the non-patterned constraint layer with 6-mm thickness had the best conformability among other grippers with 4- and 8-mm layers.

The stiffness pattern of the soft gripper was also optimized to target the star-shaped object. The actuating pressure was selected to be 30 kPa. The optimized stiffness pattern was 6, 8, 8, 4, and 4 mm, from the proximal node to the distal node. Similar to the sphere-shaped object, the thickness of the constraint layer for the non-patterned soft gripper was selected to be 6 mm, which maximized the conformability to the star-shaped object. Both the customized and non-patterned soft grippers were fabricated with the same material (Dragon Skin 30, Smooth-On Inc.). In addition, the designs of the air chamber sections were identical for both grippers.

### Comparison Between Customized and Non-patterned Grippers

To evaluate the efficiency and safety, the object pulling force and the contact forces of the soft grippers with customized stiffness patterned constraint layers and the non-patterned were compared. The contact force determines the safety of the object; a lower contact force yields a safer interaction between the object and the gripper. The pulling force determines the load capacity of the gripper; a higher pulling force under the same actuation pressure enables the gripper to grasp heavier objects. First, the object was placed at the predetermined location without actuating the gripper. Then, the soft gripper was actuated to grasp the object. The object was forced out of the gripper by slowly pulling it toward the outward direction. The pulling force of the gripper, the contact forces between the gripper and the object, and the pulling displacement were measured simultaneously. Each experiment was performed five times, and the two results with maximum and minimum values were excluded from the analysis.

#### Experimental Results for Sphere-Shaped Object

**Figure 12:**
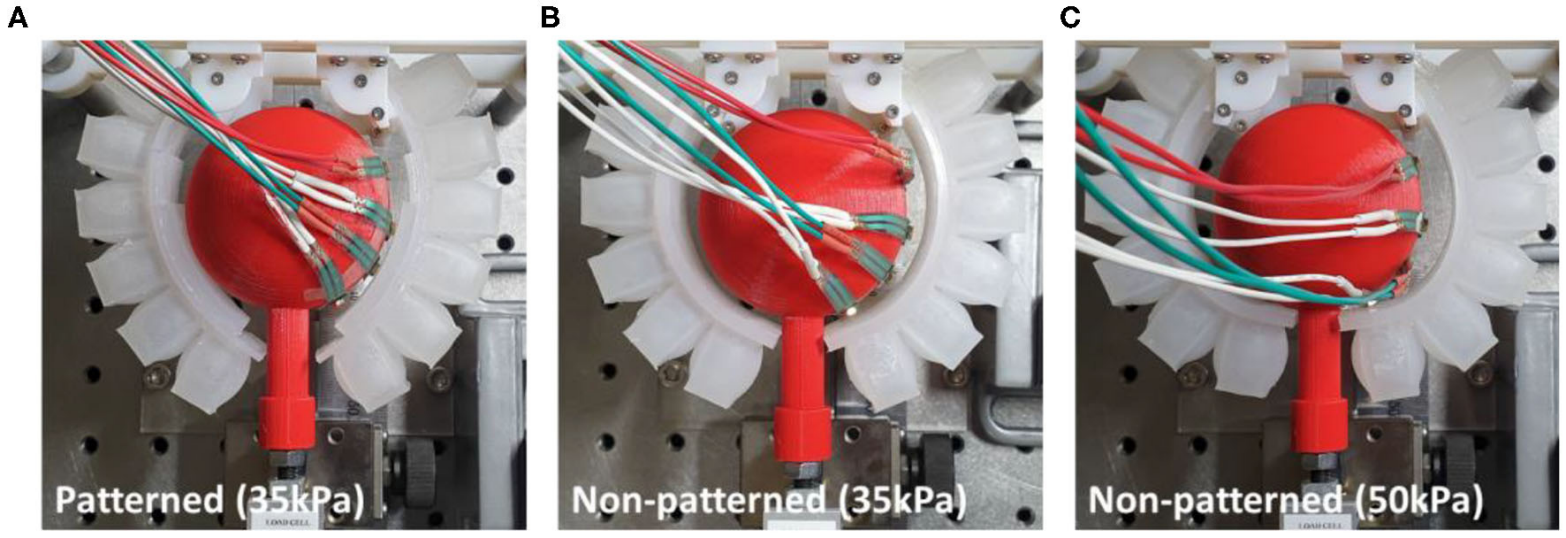
Actuated state of three cases of patterned and non-patterned soft grippers. **(A)** The soft gripper with stiffness patterned constraint layers actuated up to 35 kPa. **(B)** The non-patterned soft gripper with 35 kPa of actuation pressure. **(C)** The non-patterned soft gripper with 50 kPa of actuation pressure. Figure 13 of the supplementary section illustrates a more detailed version of the experimental results, including the sequence of grasping, and markers that correspond to the model simulations.

**Figure 13:**
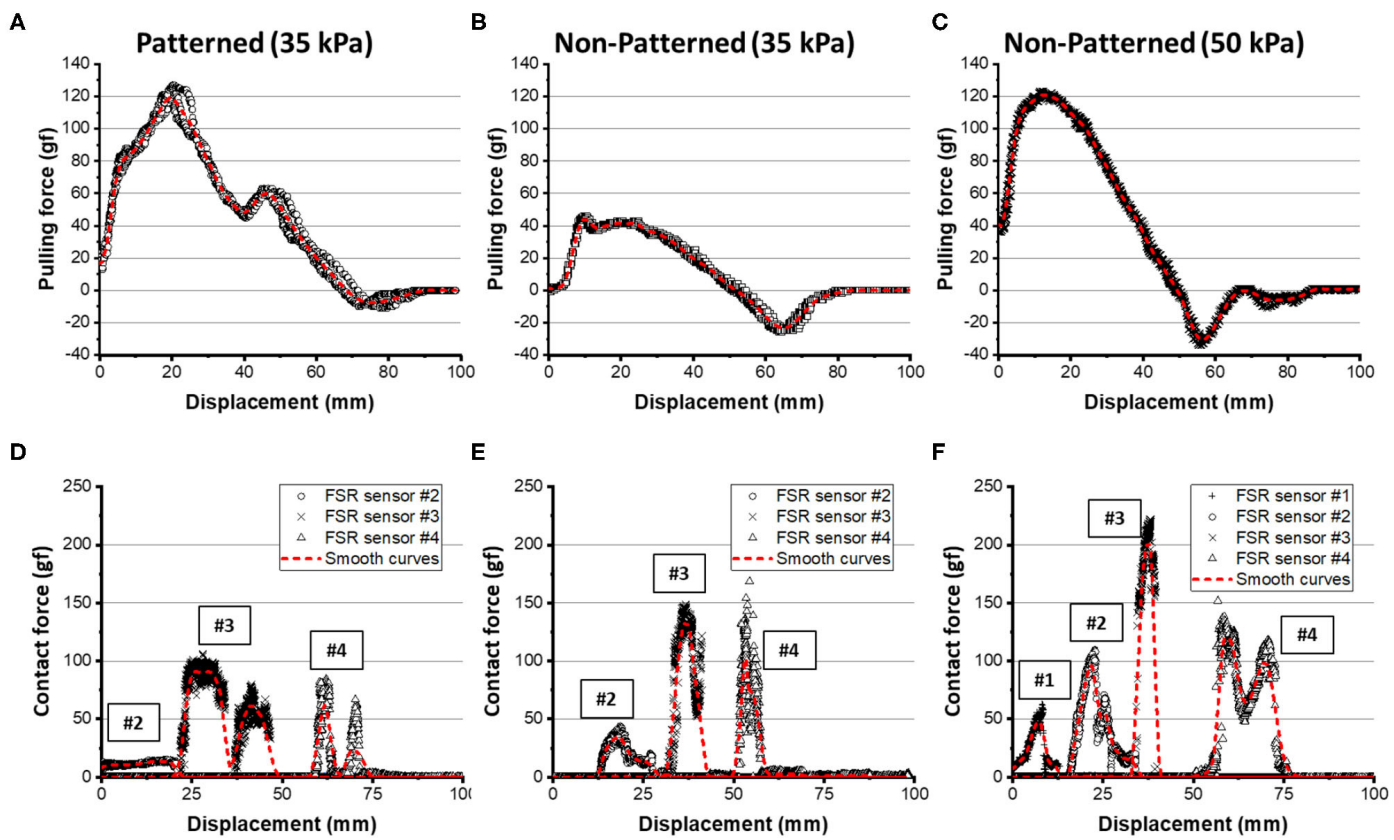
Experimental results for pulling force and contact forces on the spherical object. **(A,D)** The results for the patterned soft gripper actuated at 35 kPa. **(B,E)** The results for the non-patterned soft gripper actuated at 35 kPa. **(C,F)** The results for the non-patterned soft gripper actuated at 50 kPa. Red dashed lines are smoothed curves obtained by using a LOWESS regression with a span of 0.1.

The patterned soft gripper and the non-patterned soft gripper were actuated with the same predetermined actuating pressure, 35 kPa (Figure 12). The experimental results regarding the pulling forces for the patterned and non-patterned grippers are illustrated in Figure 13. The maximum pulling force for the patterned soft gripper is almost three times larger than that obtained for the non-patterned soft gripper under the same actuation pressure (Table 2). The non-patterned soft gripper required an actuation pressure of 50 kPa, which is approximately 1.4 times greater compared to that of the patterned soft gripper, which required 35 kPa of actuation pressure.

**Table 2:** The experimental results about grasping sphere-shaped objects.

	**Patterned (35 kPa)**	**Non-patterned (35 kPa)**	**Non-patterned (50 kPa)**
**Pulling force (experiments)**	119.12	44.03	121.05
**FSR #1**	0	0	48.22
**FSR #2**	13.76	34.28	96.16
**FSR #3**	90.89	132.54	201.67
**FSR #4**	61.84	102.60	120.45

The contact forces obtained from the four FSR sensors attached on the object's surface are illustrated in Figure 13. The customized gripper and the non-patterned gripper with 35 kPa of actuation had almost zero forces on FSR sensor #1. The contact force on sensor #2, or the patterned soft gripper, was almost constant. However, the non-patterned soft gripper actuated up to 35 kPa applied a higher contact force on sensor #2. In addition, the non-patterned soft gripper applied almost 1.5 times the force on sensors #3 and #4 than the customized gripper. Moreover, contact durations for both sensors, for the non-patterned gripper, were relatively shorter than those of the customized soft gripper.

The non-patterned soft gripper actuated up to 50 kPa exerted contact force on the FSR sensor #1 from the beginning of the experiments, unlike the previous cases. Sensors #3 and #4, in this case, were applied with almost twice the maximum contact forces than those of the customized soft gripper. Moreover, the position of the sensor #4 is opposite to the lifting direction of the object.

#### Experimental Results for a Star-Shaped Object

**Figure 14:**
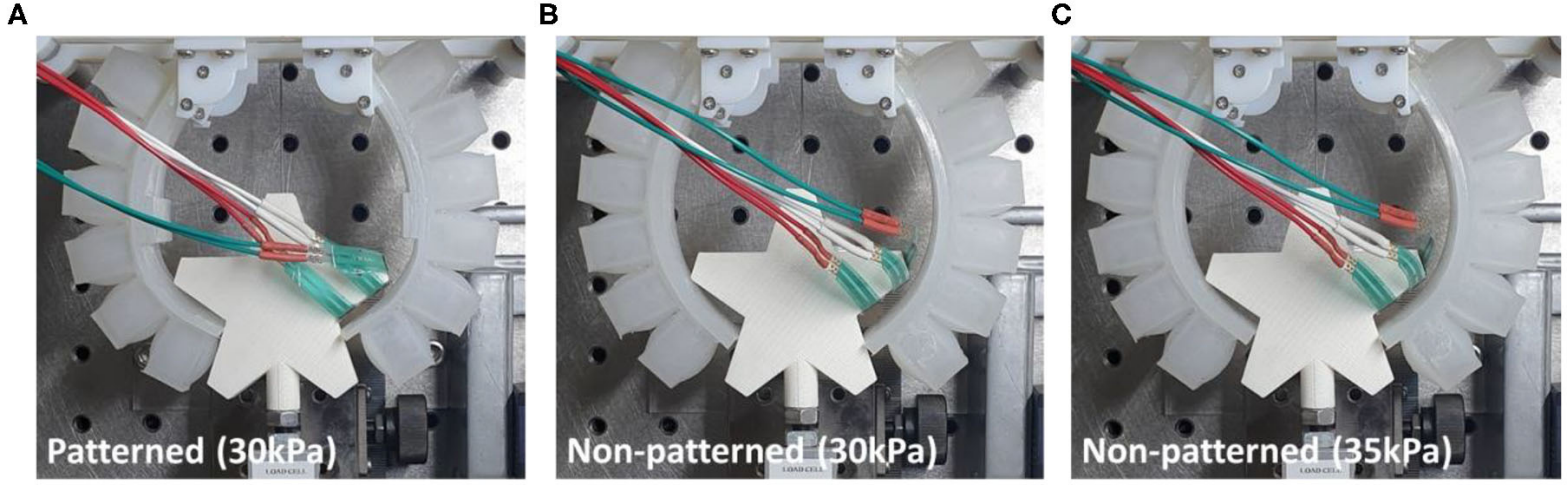
Actuated state of three cases of patterned and non-patterned soft grippers. **(A)** The soft gripper with stiffness patterned constraint layers actuated up to 30 kPa. **(B)** The non-patterned soft gripper with 30 kPa of actuation pressure. **(C)** The non-patterned soft gripper with 35 kPa of actuation pressure. Figure 13 of the supplementary section illustrates a more detailed version of the experimental results, including the sequence of grasping, and markers that correspond to the model simulations.

**Figure 15:**
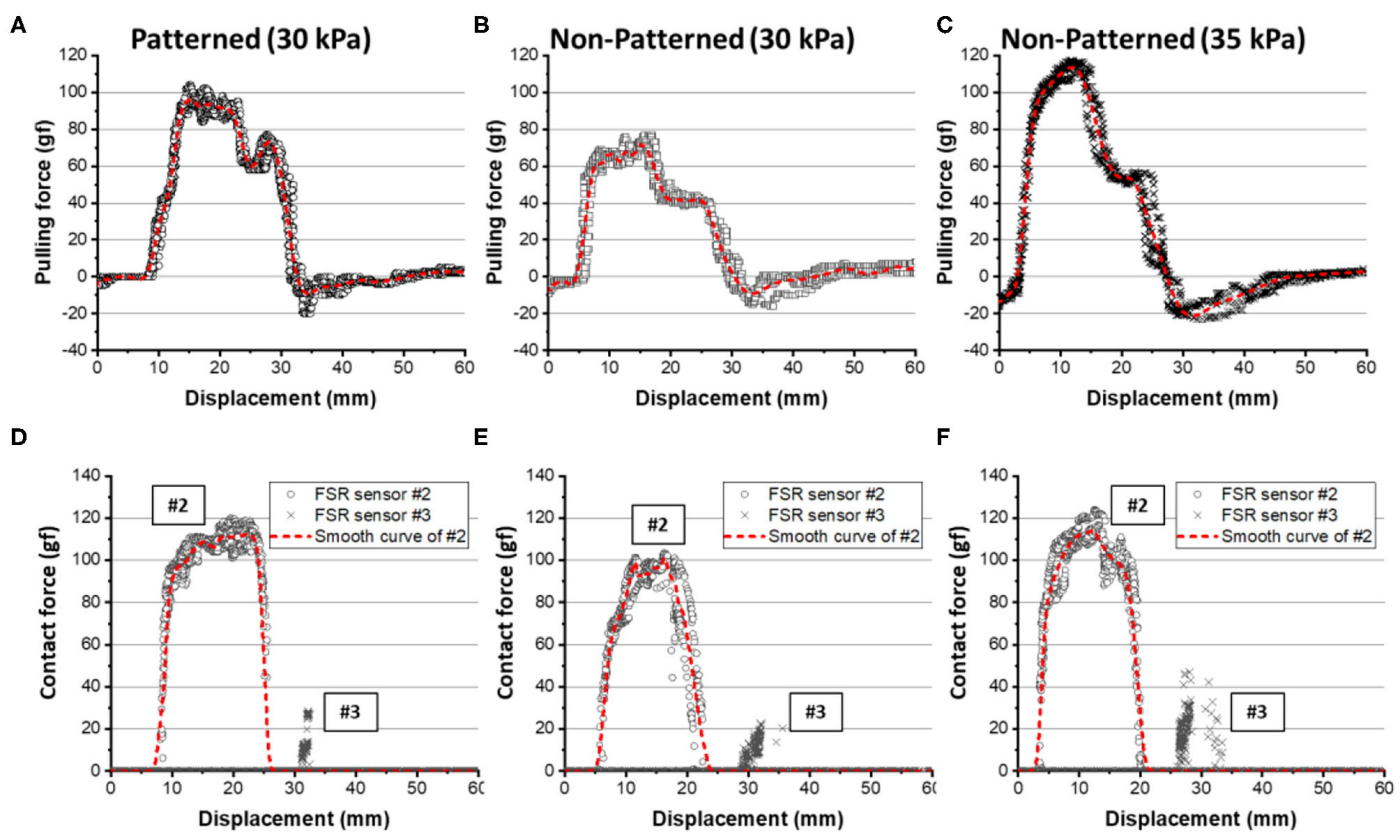
Experimental results for pulling force and contact forces on the star-shaped object. **(A,D)** The results for the patterned soft gripper actuated at 30 kPa. **(B,E)** The results for the non-patterned soft gripper actuated at 30 kPa. **(C,F)** The results for the non-patterned soft gripper actuated at 35 kPa. The red dashed lines are smoothed curves obtained by using a LOWESS regression with a span of 0.1.

The patterned and non-patterned soft grippers both grasped the star-shaped object with 30 kPa of actuating pressure (Figure 14). The patterned soft gripper had about 1.3 times the pulling force than the non-patterned soft gripper (Figure 15). The non-patterned soft gripper actuated with 35 kPa of pressure had slightly higher pulling force than the patterned soft gripper with 30 kPa of pressure (Table 3).

**Table 3:** The experimental results about grasping star-shaped objects.

	**Patterned (30 kPa)**	**Non-patterned (30 kPa)**	**Non-patterned (35 kPa)**
**Pulling force (experiments)**	95.98	71.81	113.44
**FSR #1**	–	–	–
**FSR #2**	112.45	101.20	114.01
**FSR #3**	28.25	31.65	46.95

The contact force applied on the FSR sensor #1 was almost zero, which were out of the measuring range of the sensor, for all three cases. The maximum contact force applied on FSR sensor #2 was almost similar to both for the stiffness patterned and non-patterned soft grippers. However, the contact duration was longer with the stiffness patterned soft gripper. Meanwhile, the non-patterned soft gripper with 35 kPa of actuating pressure exerted a higher contact force on the sensor #3, which was attached to the surface that is opposite to the lifting direction of the object (Figure 15).

In summary, the experimental results show that the shape-conformable soft grippers with customized constraint layer stiffness pattern designs had better grasping performance in terms of object pulling and contact forces. The stiffness patterned soft gripper may exhibit better stability and require lower actuation pressure. Furthermore, the contact forces, which may be related to the integrity of the interaction between the gripper and the object, decreased for the shape-conformable soft gripper due to stiffness patterning of the constraint layers.

## Conclusion

In this paper, we presented an analytical approach that allows us to estimate and experimentally implement customized postures of soft pneumatic grippers. The model suggested that the moment surfaces generated in the air chamber section and the constraint layer section correspond to the bending behavior of soft grippers. The computation speed of the model was relatively fast than that of numerical methods, which have been mainly used in existing studies of soft robots. Therefore, it was possible to obtain the optimal converged postures with rapid iterations for given outlining shapes of target objects. Stiffness patterning of the constraint layers of soft grippers was proposed as a facile and powerful methodology to tune the moment surfaces, in conjunction with suitable fabrication methods. Experimental results about the grasping of objects with different shapes showed that the customized grasping posture effectively reduces the contact force and the actuating pressure while maintaining the lifting force.

Future works include enhancing the proposed analytical model and further developing the customization approach. The proposed analytical model requires experimental results regarding the single air chamber inflation test and the three-point bending test. However, the results, obtained from the numerical analysis, such as finite-element analysis, can replace experimental results of the model. Ultimately, the model can be expanded into a hybrid framework that uses the rapid computing speeds of the analytical approach and the preciseness of the numerical method. Implementing topological optimization methodologies into the constraint layer can provide smooth transitions of stiffness profiles that establish grasping postures with better conformability to target objects. Furthermore, the rapid computing speeds of the analytical model can be utilized to generate an abundance of data for machine learning-based optimization processes.

Finally, with our grasping posture customization approach, we hope that soft grippers would take a step closer to the industrial scenes.

## Data Availability Statement

The original contributions presented in the study are included in the article/Supplementary Material, further inquiries can be directed to the corresponding author.

## Author Contributions

J-YL built the analytical model, planned and performed the experiments, and also was the main writer of the manuscript. JE performed experiments, supported building the model, and supported the preparation of the manuscript. SY performed experiments and supported the preparation of the manuscript. KC reviewed and guided the proposed analytical model, experimental plans, and the preparation of the manuscript. All authors have contributed to the article and approved the submitted version.

## Conflict of Interest

The authors declare that the research was conducted in the absence of any commercial or financial relationships that could be construed as a potential conflict of interest.

## References

[B1] Al Abeach L. A. T. Nefti-Meziani S. Davis S. (2017). Design of a variable stiffness soft dexterous gripper. Soft Robot. 4, 274–284. 10.1089/soro.2016.0044 29062630 PMC5649399

[B2] Amend J. R. Brown E. M. Rodenberg N. Jaeger H. M. Lipson H. (2012). A Positive Pressure Universal Gripper Based on the Jamming of Granular Material. IEEE Trans. Robot. 28, 341–350. 10.1109/TRO.2011.2171093

[B3] Cheng N. Amend J. Farrell T. Latour D. Martinez C. Johansson J.. (2016). Prosthetic jamming terminal device: a case study of untethered soft robotics. Soft Robot. 3, 205–212. 10.1089/soro.2016.0017 28078196 PMC5180076

[B4] Cheng N. G. Gopinath A. Wang L. Iagnemma K. Hosoi A. E. (2014). Thermally tunable, self-healing composites for soft robotic applications. Macromol. Mater. Eng. 299, 1279–1284. 10.1002/mame.201400017

[B5] Dollar A. M. Howe R. D. (2006). A robust compliant grasper via shape deposition manufacturing. IEEE/ASME Trans. Mechatron. 11, 154–161. 10.1109/TMECH.2006.871090

[B6] Fei Y. Wang J. Pang W. (2018). A novel fabric-based versatile and stiffness-tunable soft gripper integrating soft pneumatic fingers and wrist. Soft Robot. 6, 1–20. 10.1089/soro.2018.0015 30312144

[B7] Glick P. Suresh S. A. Ruffatto D. Cutkosky M. Tolley M. T. Parness A. (2018). A soft robotic gripper with gecko-inspired adhesive. IEEE Robot. Autom. Lett. 3, 903–910. 10.1109/LRA.2018.2792688

[B8] Gorissen B. Reynaerts D. Konishi S. Yoshida K. Kim J. W. De Volder M. (2017). Elastic inflatable actuators for soft robotic applications. Adv. Mater. 29:1604977. 10.1002/adma.201604977 28949425

[B9] Hao Y. Wang T. Xie Z. Sun W. Liu Z. Fang X. (2018). A eutectic-alloy-infused soft actuator with sensing, tunable degrees of freedom, and stiffness properties. J. Micromech. Microeng. 28:024004 10.1088/1361-6439/aa9d0e

[B10] Howell L. L. (2001). Compliant Mechanisms. Hoboken, NJ: John Wiley & Sons.

[B11] Hu W. Li W. Alici G. (2018). 3D printed helical soft pneumatic actuators. In: 2018 IEEE/ASME International Conference on Advanced Intelligent Mechatronics (AIM) (Auckland), 950–955. 10.1109/AIM.2018.8452456

[B12] Hughes J. Culha U. Giardina F. Guenther F. Rosendo A. Iida F. (2016). Soft manipulators and grippers: a review. Front. Robot. AI 3:69 10.3389/frobt.2016.00069

[B13] Hurtado J. F. Melkote S. N. (2001). Effect of conformability on grasp static stability, in Proceedings 2001 ICRA. IEEE International Conference on Robotics and Automation (Cat. No.01CH37164) (Seoul), 1086–1091.

[B14] Lee J. Y. Eom J. Choi W. Y. Cho K. J. (2018). Soft LEGO: bottom-up design platform for soft robotics. In 2018 IEEE/RSJ International Conference on Intelligent Robots and Systems (IROS) (Madrid), 7513–7520. 10.1109/IROS.2018.8593546

[B15] Lee J. Y. Kim W. B. Choi W. Y. Cho K. J. (2016). Soft robotic blocks: introducing SoBL, a fast-build modularized design block. IEEE Robot. Autom. Mag. 23, 30–41. 10.1109/MRA.2016.2580479

[B16] MacCurdy R. Katzschmann R. Kim Y. Rus D. (2016). Printable hydraulics: a method for fabricating robots by 3D Co-printing solids and liquids. Maccurdy. Available online at: http://dspace.mit.edu/handle/1721.1/103072 (accessed January 9, 2019). 10.1109/ICRA.2016.7487576

[B17] Milana E. Gorissen B. Voider M. D. Reynaerts D. (2018). Design of a bi-segmented soft actuator with hardware encoded quasi-static inflation sequence. In: 2018 IEEE International Conference on Soft Robotics (RoboSoft) (Livorno), 108–113. 10.1109/ROBOSOFT.2018.8404905

[B18] Mutlu R. Tawk C. Alici G. Sariyildiz E. (2017). A 3D printed monolithic soft gripper with adjustable stiffness. In: IECON 2017 - 43rd Annual Conference of the IEEE Industrial Electronics Society (Beijing), 6235–6240. 10.1109/IECON.2017.8217084

[B19] Park W. Seo S. Bae J. (2019). A hybrid gripper with soft material and rigid structures. IEEE Robot. Autom. Lett. 4, 65–72. 10.1109/LRA.2018.2878972

[B20] Pauly J. Midha A. (2006a). Pseudo-Rigid-Body Model Chain Algorithm: Part 1 — Introduction and Concept Development, in ASME 2006 International Design Engineering Technical Conferences and Computers and Information in Engineering Conference (Philadelphia, PA), 173–181. 10.1115/DETC2006-99460

[B21] Pauly J. Midha A. (2006b). Pseudo-Rigid-Body Model Chain Algorithm: Part 2 — Equivalent Representations for Combined Load Boundary Conditions, in ASME 2006 International Design Engineering Technical Conferences and Computers and Information in Engineering Conference (Philadelphia, PA), 183–190. 10.1115/DETC2006-99463

[B22] Rus D. Tolley M. T. (2015). Design, fabrication and control of soft robots. Nature 521, 467–475. 10.1038/nature14543 26017446

[B23] Shian S. Bertoldi K. Clarke D. R. (2015). Dielectric elastomer based “grippers” for soft robotics. Adv. Mater. 27, 6814–6819. 10.1002/adma.201503078 26418227

[B24] Shimoga K. B. Goldenberg A. A. (1992). Soft materials for robotic fingers. In: Proceedings 1992 IEEE International Conference on Robotics and Automation (Nice), 1300–1305. 10.1109/ROBOT.1992.220069

[B25] Shintake J. Cacucciolo V. Floreano D. Shea H. (2018). Soft robotic grippers. Adv. Mater. 30:1707035 10.1002/adma.201707035 29736928

[B26] Shintake J. Sonar H. Piskarev E. Paik J. Floreano D. (2017). Soft Pneumatic Gelatin Actuator for Edible Robotics. *arXiv:1703.01423 [cs]*. Available online at: http://arxiv.org/abs/1703.01423 (accessed March 14, 2017). 10.1109/IROS.2017.8206525

[B27] Su Y. Fang Z. Zhu W. Sun X. Zhu Y. Wang H. (2020). A high-payload proprioceptive hybrid robotic gripper with soft origamic actuators. IEEE Robot. Autom. Lett. 5, 3003–3010. 10.1109/LRA.2020.2974438

[B28] Terryn S. Brancart J. Lefeber D. Assche G. V. Vanderborght B. (2017). Self-healing soft pneumatic robots. Sci. Robot. 2:eaan4268. 10.1126/scirobotics.aan4268 33157852

[B29] Terryn S. Mathijssen G. Brancart J. Lefeber D. Assche G. V. Vanderborght B. (2015). Development of a self-healing soft pneumatic actuator: a first concept. Bioinspir. Biomim. 10:046007. 10.1088/1748-3190/10/4/046007 26151944

[B30] Wei Y. Chen Y. Ren T. Chen Q. Yan C. Yang Y. (2016). A novel, variable stiffness robotic gripper based on integrated soft actuating and particle jamming. Soft Robot. 3, 134–143. 10.1089/soro.2016.0027

[B31] Zhang H. Kumar A. S. Chen F. Fuh J. Y. H. Wang M. Y. (2018). Topology optimized multimaterial soft fingers for applications on grippers, rehabilitation and artificial hands. IEEE/ASME Trans. Mechatron. 24, 120–131. 10.1109/TMECH.2018.2874067

[B32] Zhou J. Chen S. Wang Z. (2017). A soft-robotic gripper with enhanced object adaptation and grasping reliability. IEEE Robot. Autom. Lett. 2, 2287–2293. 10.1109/LRA.2017.2716445

