# Supplementary material for: Customization Methodology for Conformable Grasping Posture of Soft Grippers by Stiffness Patterning
Source: Front Robot AI. 2020 Sep 18;7:114. doi: 10.3389/frobt.2020.00114 (PMC7805940; doi:10.3389/frobt.2020.00114)
Supplement: Supplementary file 1 [file Data_Sheet_1.pdf]

Supplementary Material

1 Contents of the Supplementary Material

This Supplementary Material contains the following contents. Section 2 presents the experimental results of customized soft grippers, which were fabricated with variable thickness mold and modular design methods. The experimental results include the comparisons between the experimental results and the analytical model's estimations regarding the soft finger's actuated postures. In addition, details regarding the experiments for building the analytical model, such as air chamber inflation and bending stiffness of constraint layers are also presented in Section 2.

In the following section, Section 3, the analytical model is applied in estimating grasping postures of soft grippers when they are in contact with target objects. Experimental results and a pseudo-code for the algorithm are also presented in Section 3.

2 Physical Demonstration of Stiffness Patterning

In this chapter, the results of the physical demonstrations of soft grippers with stiffness patterning of the constraint layers will be shown for two fabrication methods. One is patterning stiffness by changing the thickness of the constraint layers. The other approach uses different materials for each section of the layer. Details regarding fabrication methods are presented in the main article. In this Supplementary Material, the experimental results and their comparisons to the analytical model's estimations are presented.

2.1 Stiffness Patterning with Variable Thickness Mold

Most soft robots have been fabricated using elastomeric polymers molding methods. We propose using a variable thickness mold for molding-based fabrication of stiffness patterned constraint layers (Supplementary Figure 1). Constraint layers with different stiffness patterns can be fabricated using a single reusable mold set. A variable thickness mold is proposed as an approach to change the thickness to reduce manufacturing costs and time.

The reusable and variable thickness mold has two components: a base mold and thickness tuning plates. The thickness tuning plates are inserted into the base mold to modify the stiffness pattern of the constraint layer. Which means a new mold isn't required to modify the stiffness pattern of the constrain layer. The mold can be modified by changing the configuration of the thickness tuning plates, as shown in Supplementary Figure 2. The variable thickness mold has advantages over the material changing method when patterning stiffness of the layer because the constraint layer could be fabricated with a single molding process. Although, different stiffness patterns can be obtained by using multiple materials, pouring and curing processes for different materials may need multiple steps which lengthens the fabrication time.

We tested three different stiffness levels (thickness of 4, 6, and 8 mm). The dimensions of a soft finger with a non-patterned constraint layer are presented in Supplementary Figure 1. The thickness of the mold was 2 mm thinner than the designed thickness of the constraint layer (t_{layer}) because the demolded air chamber section already had a 2 mm thin shell at the bottom surface to which the constraint layer is attached.

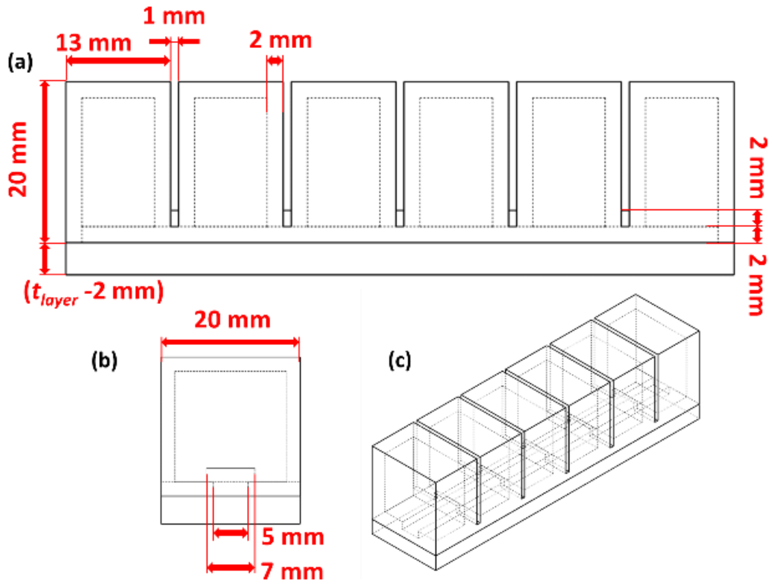

Supplementary Figure 1. Dimensions of the soft finger with the non-patterned constraint layer. (a) Side view of the finger. (b) Frontal view of the finger. (c) Isometric view of the finger. All views, (a), (b), and (c) with the hidden lines.

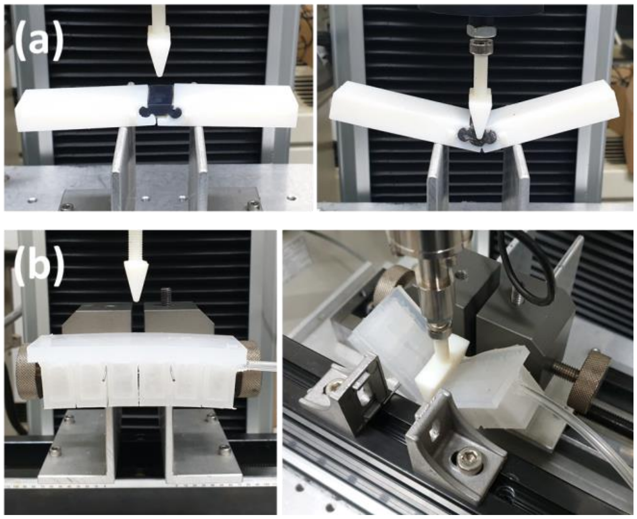

Supplementary Figure 2. The moments generated by the constraint layers were obtained using three-point bending tests. (a) Test procedure for the flexure block of the modularized design of soft grippers (b) Test procedure for the soft gripper by elastomer molding method.

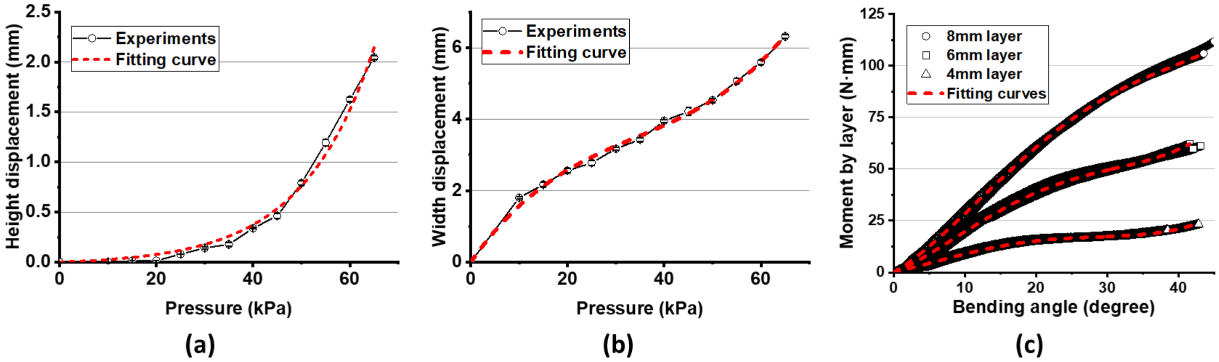

Supplementary Figure 3. **Experimental results for the single air chamber inflation test and the three-point bending tests of the constraint layers. (a) Height displacement during air chamber inflation. (b) Width displacement during air chamber inflation. (c) Three-point bending test of the constraint layers for three different thicknesses.**

The simplified analytical model requires experimental data regarding the width and height displacements of a single air chamber during inflation (d_{height}, d_{width}) and the moment generated by the constraint layer (M_{layer}). The inflation behavior of the single air chamber was captured by video and subsequently analysed. The moments generated by the constraint layers were obtained from the three-point bending tests (Figure 15). Using appropriate fitting curves, equations (S1), (S2) and (S3), were imported into the simplified model. Each of the three samples was tested twice.

$$d_{height} = -0.028 \cdot \left(1 - e^{(-0.067 \cdot p)}\right) \qquad \text{(S1)}$$

$$d_{width} = 0.184 \cdot p - 0.036 \cdot p^2 + 3.399 \times 10^{-5} \cdot p^3 \qquad \text{(S2)}$$

$$M_{layer} = \begin{cases} 70.88 \cdot \theta - 11.70 \cdot \theta^2 - 39.91 \cdot \theta^3 + 24.73 \cdot \theta^4 & (t_{layer} = 4\,mm) \\ 88.08 \cdot \theta + 147.0 \cdot \theta^2 - 209.1 \cdot \theta^3 + 72.79 \cdot \theta^4 & (t_{layer} = 6\,mm) \\ 72.45 \cdot \theta + 312.3 \cdot \theta^2 - 306.5 \cdot \theta^3 + 86.65 \cdot \theta^4 & (t_{layer} = 8\,mm) \end{cases} \qquad \text{(S3)}$$

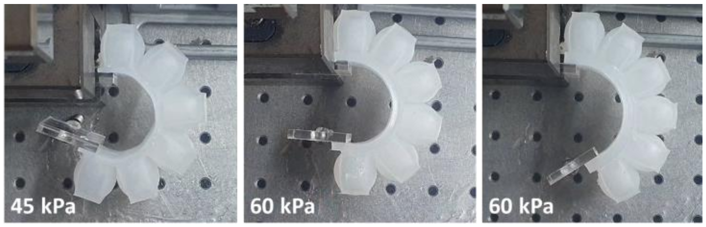

(a) (b) (c)

Supplementary Figure 4. **Three soft fingers with different constraint layers.**

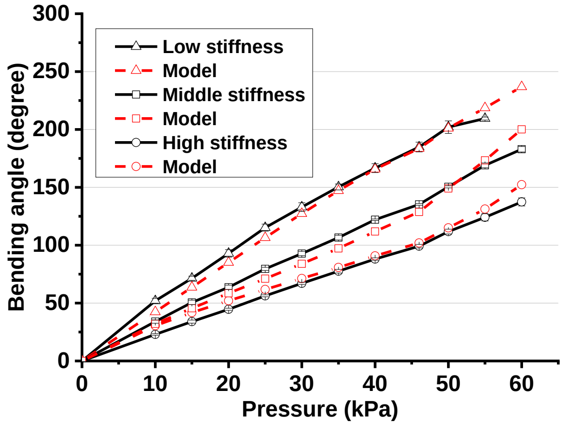

Supplementary Figure 5. Experimental results regarding the pressure and bending angles. Red dashed lines indicate results from simulations. Error bars indicate standard error of mean. Error bars represent standard errors, and marks represent mean values.

Soft fingers with three different thickness were tested (Supplementary Figure 5). The soft finger with the softest layer (4 mm) was actuated up to 40 kPa because higher pressures resulted in the fingertip contacting the basement. The experimental results were compared with simulated results in Supplementary Figure 6. The errors between the model and experiments may have been caused by the nonlinear distortion of the soft fingers during actuation. The stiffness patterned actuator was also tested (Supplementary Figure 6). Compared to the non-patterned actuator, the patterned actuator had irregular bending curvatures in the longitudinal direction.

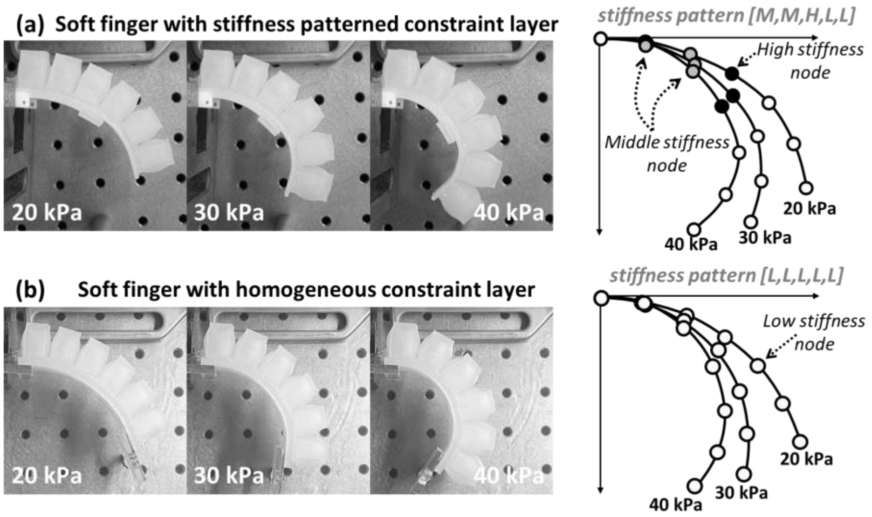

Supplementary Figure 6. Comparison between non-uniform stiffness patterned soft finger and uniform stiffness soft finger. Right schematic diagram shows the shape of each soft finger at a given pressure. 'L', 'M', and 'H' represent the low stiffness (white), middle stiffness (grey), and high stiffness (black) patterned nodes, respectively.

2.2 Stiffness Patterning with a Modular Design

In this subchapter, the modularized design for soft grippers is introduced. Based on our previous researches, the design of the modules was revised to implement the stiffness patterning concept. The module block set for stiffness patterning has three main parts; an air chamber block, bottom blocks, and flexure blocks (Supplementary Figure 8). They were fabricated via 3-dimenstional printing (Connex 260, Stratasys Inc.). The inflatable air chamber block was built using both the rubber-like soft material (TangoBlack+) and the ABS-like stiff material (VeroWhite+). The bottom blocks were built with only the ABS like material. Stiffness of the flexure blocks was divided in three levels with three different materials (TangoBlack+, FLX9840-DM, FLX9860-DM, Stratasys Inc.). All the blocks were reusable and easy to assemble and dismantle.

The flexure blocks connect the bottom blocks to form the bending block. Both the flexure block and bottom the blocks have an air channel in the middle of their structure. Therefore, they are also pneumatically connected. The flexure block, which acts as the bottom layer of the elastomeric polymer-based soft bending actuators, was designed to be exchangeable. Therefore, the user could change the configuration of the stiffness pattern of the bending block by changing the flexure blocks (Details are presented Figure 11 in main article). When the bending block are bent by actuation, the zig-zag shaped slits on the top surface contacts each other. The center of rotation was placed on the contact points which means the flexure block only experiences compression. This design prolongs the life of the flexure block.

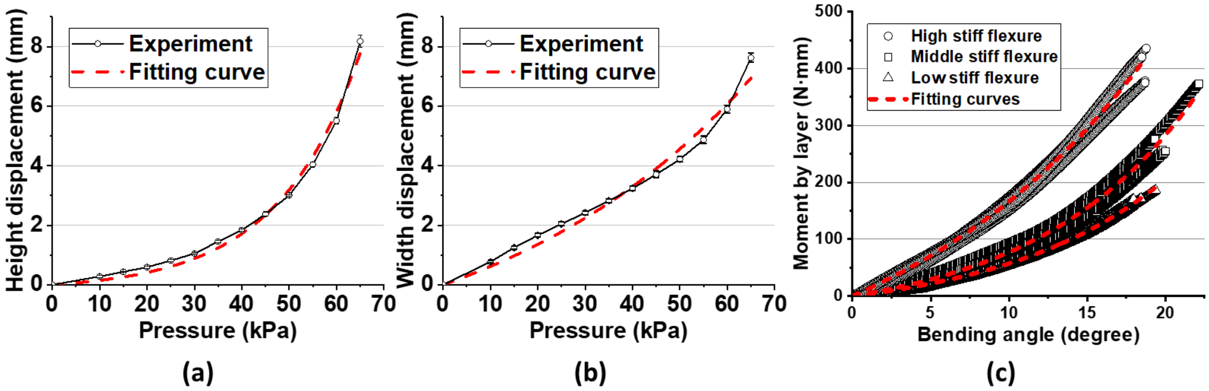

Supplementary Figure 7. Height and width displacements of the singe air chamber and the three-point bending tests for modular design. (a) Height displacement of the single air chamber block. (b) Width displacement of the single air chamber block. (c) Three-point bending tests for three kinds of flexure blocks with different stiffness.

Inflating behaviors of the single air chamber block and the moment generated by the constraint layers with different flexure blocks were tested, like the elastomer molded soft fingers (Supplementary Figure 7). The fitting curves from experimental results (Equation (S4) to (S6)) were implemented into the analytical model for posture estimation.

$$d_{height} = -0.183 \cdot \left(1 - e^{(-0.058 \cdot p)}\right) \quad \text{(S4)}$$

$$d_{width} = -2.680 \cdot \left(1 - e^{(-0.020 \cdot p)}\right) \quad \text{(S5)}$$

$$M_{layer} = \begin{cases} -66.28 \cdot \left(1 - e^{(-2.833 \cdot p)}\right) & (Low\ stiffness) \\ -92.32 \cdot \left(1 - e^{(-2.814 \cdot p)}\right) & (Middle\ stiffness) \\ -410.1 \cdot \left(1 - e^{(-1.702 \cdot p)}\right) & (High\ stiffness) \end{cases} \quad \text{(S6)}$$

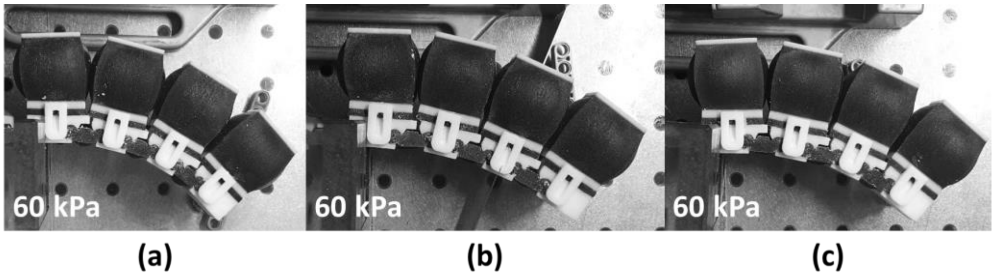

Supplementary Figure 8. **Three different kinds of soft fingers actuated at 60 kPa. (a), (b), and (c) are results of the soft grippers with the softest flexure, medium flexure, and stiffest flexure, respectively.**

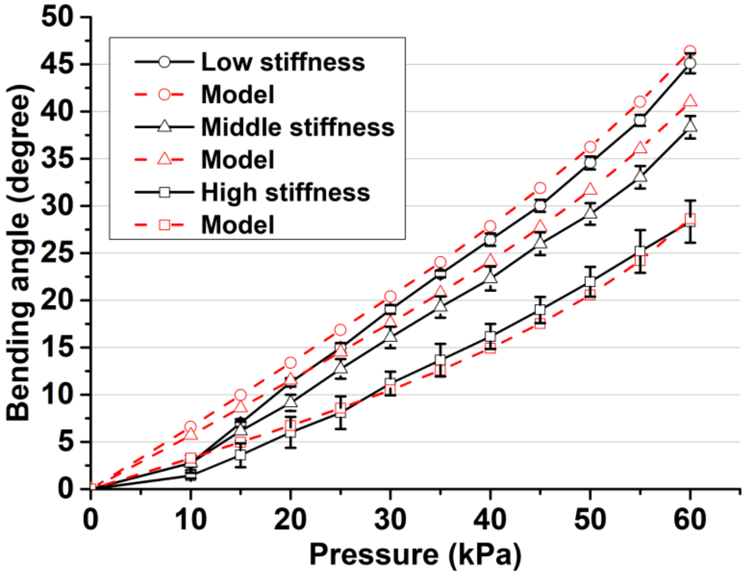

Supplementary Figure 9. **Experimental results of the pressure and bending angles. Red dashed lines indicate results from simulations. Error bars indicate standard error of mean. Error bars represent standard errors, and marks represents mean values.**

Supplementary Figure 8 shows the flexure of soft fingers with different actuated states. Supplementary Figure 9 shows experimental results regarding the pressure and bending angles of three different kinds of actuators assembled with low, middle, and high stiffness flexures. The simplified model matches the experimental results relatively well compared to that of elastomer molding-based soft actuators. Relatively large errors occur under 20 kPa of pressure. This error may cause by the gap between slits on the bottom blocks' top surface.

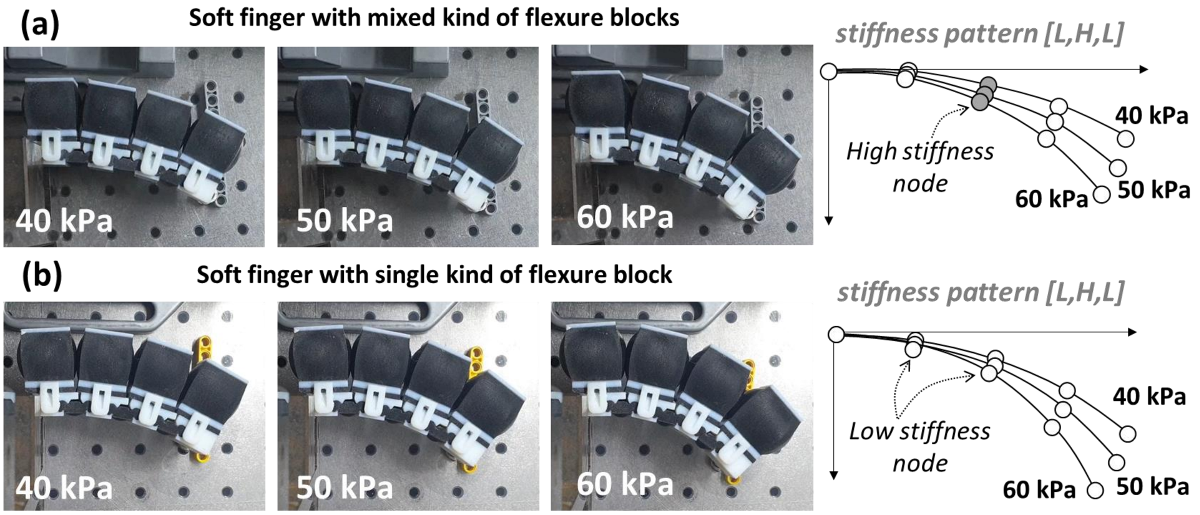

Supplementary Figure 10. Comparison between non-uniform stiffness patterned soft finger and uniforms stiffness soft finger. Right schematic diagram shows the shape of each soft finger at certain pressures. 'L' means low stiffness pattern node and 'H' means high stiffness node.

The stiffness patterned soft actuator was also assembled and tested (Supplementary Figure 10). The flexure block at the center of the soft finger was switched from the softest block to the high stiffness block. Therefore, the curvature at the center of the soft finger was smaller than that of the other sections. However, the bending angle differences between different kinds of flexure blocks were hardly noticeable. One way to enlarge a difference is to put the notch design on the bottom of the flexure blocks.

3 Grasping Posture Estimation with The Analytical Model

3.1 Estimating Grasping Posture and Experimental Results

It is challenging to estimate the behavior of the soft bending actuator when the structure contacts or interacts with the external environment. The contacted posture of a soft bending actuator could be estimated with the pseudo-rigid-body model chain algorithm (Pauly and Midha, 2006a). Equation (S7), which is the governing moment equivalent equation for the *i*-th node of a soft bending actuator with a contact force, could be derived from the PRBM chain algorithm. The moment due to the chamber and the moment due to the layer are functions of the bending angle and actuation pressure.

The actuation pressure is given, therefore the bending angle could be obtained by solving equation (S7).

$$M_{chamber,i} = M_{layer,i} + \sum_{k=i}^{n}(\vec{r}_{i,k} \times \vec{f}_{contact,k}) \qquad \text{(S7)}$$

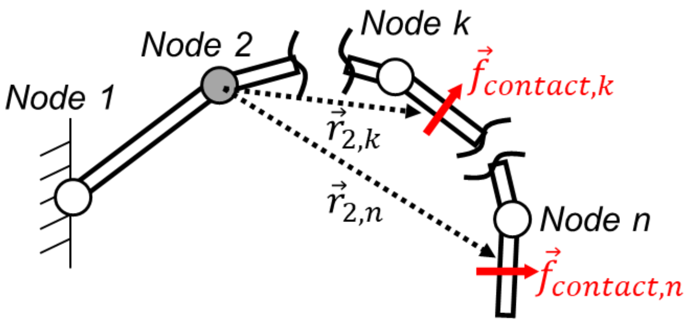

Supplementary Figure 11. Schematic diagram for moment calculation by contact forces at the second node.

Supplementary Figure 11 shows how the moments generated by contact forces can be calculated using the pseudo-rigid-body model chain algorithm. We assumed that a contact force was applied to an element that overlapped with a grasping object. It was also assumed that a contact point of the *k*-th element was applied at the center of the overlapped area between the element and the object. The position vector between the *i*-th node and the contact point of the *k*-th element, $\vec{r}_{i,k}$, can be obtained under these assumptions. Deformation caused by frictional force applied on the soft gripper was neglected. The load increment technique for the PRBM chain algorithm was implemented into the estimating algorithm to reduce errors.

Grasping postures of the soft grippers were estimated through the proposed analytical model. The chain algorithm for the pseudo-rigid-body model was implemented to the analytical model for estimating the postures of the soft grippers (Supplementary Algorithm 1). In recent efforts, models for discrete air chamber type actuators were introduced (Alici et al., 2017; Natividad et al., 2018). However, these models do not consider the interactions between the target objects and the gripper when estimating the actuators' bending angles.

The schematic diagram in Supplementary Figure 12 shows the algorithm for estimating a grasping posture of a soft gripper with contacts. First, the gripper was actuated by the given pressure input. A target object was placed at an initial position. Then the elements and nodes of the gripper, which overlap to the object, are found. It was assumed that the contact forces were located at the middle of the overlapped area of each element. Each contact force was inversely found depending on the weight of the applied. It was assumed that the contact forces are distributed proportionally to the contact angle between the element and the object. The coefficient of friction was measured to be 0.78, under the same weight load as the measured lifting force, while considering the surface texture of 3-D printed objects. The applied weight of the object is increased for each step by using the load increment technique (Pauly and Midha, 2006a). The grasping posture estimating algorithm is terminated when the opening of the gripper is wider than the maximum width of the object.

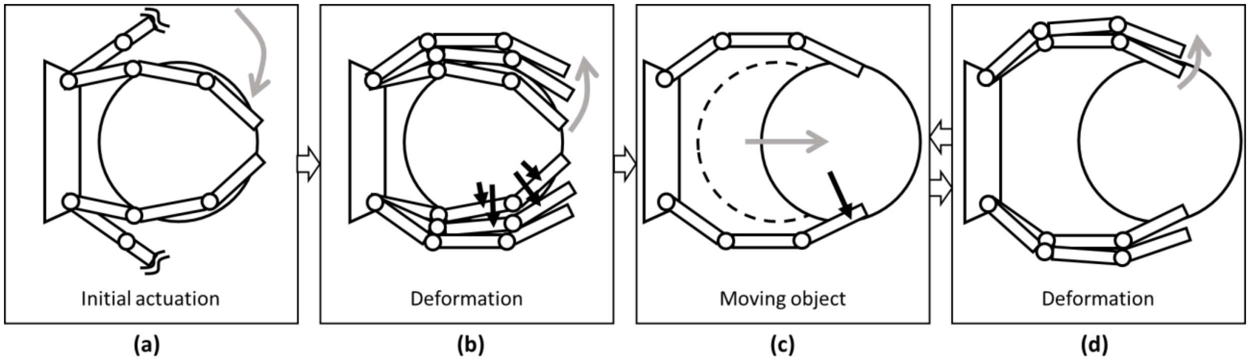

Supplementary Figure 12. Overview of the grasping posture estimating algorithm. (a) The gripper actuated without any contact. (b) Finding contact points and contact forces and estimating deformed postures with the proposed analytical model and the load increment technique. (c) Moving the object with small steps until there is an overlap. (d) Repeat until the opening of the gripper is wider than the object.

The experimental results and the estimated postures computed by the simplified analytical model were compared in Supplementary Figure 13. The simplified analytical model could estimate deformations of the grippers within tens of seconds, which is relatively fast than the numerical analysis methods.

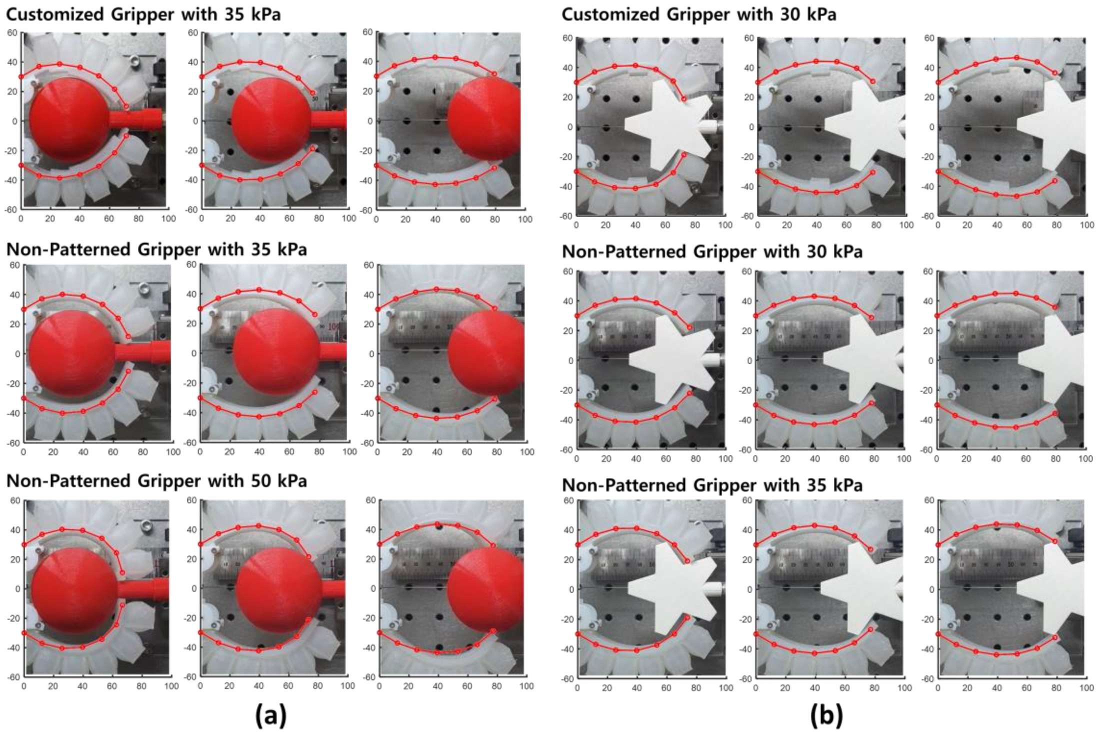

Supplementary Figure 13. Comparison between experimental results and simulated posture of the soft grippers. (a) Grasping the sphere-shaped object. (b) Grasping the star-shaped object.

3.2 Algorithm for Estimating Grasping Posture

Supplementary Algorithm 1 represents the pseudo code for the analytical model that estimates grasping postures of customized soft grippers.

Supplementary Algorithm 1. Estimating posture of soft bending actuator with contacts.

Input: p_{input} // Actuating pressure

$S = [s_1, s_2, ..., s_n]$ // Stiffness pattern of the soft gripper with m numbers of nodes

$F = [\boldsymbol{f_1}, \boldsymbol{f_2}, ..., \boldsymbol{f_n}]$ // Contact forces for each element

N_{step} // Numbers of load increments

Output: $G_{shape} = [\theta_1, \theta_2, ..., \theta_n]$ // Posture of the soft finger represented by a bending angle of each node

1: G_{shape} = PostureEstimator(p_{input}, S); // Get initial posture without contact

2: **for** $i := 1$ **to** N_{step} **Do**

2: **for** $j := 1$ **to** n **Do**

$M_{contact,j} \leftarrow 0$; // Initialize moment generated by contact for current step

3: **for** $k := j$ **to** n **Do**

$\boldsymbol{r_{j,k}} \leftarrow$ PositionVector(G_{shape}); // Get position vector from j-th node to contact point of k-th element

$\boldsymbol{f_{k,step}} \leftarrow i \cdot \boldsymbol{f_k}/N_{step}$; // Get force applied on this step with the load increment technique

$M_{contact,j} \leftarrow M_{contact,j} + \boldsymbol{r_{j,k}} \times \boldsymbol{f_{k,step}}$; // Update the moment generated by forces

end for

4: **Find** θ_j **that**

5: $M_{chamber,j}(\theta_j, p_{input}) = M_{layer,j}(\theta_j, s_j) + M_{contact,j}$; // Solve the equation about moments generated by chambers and layer at *i*-th node and contact forces

G_{shape} = ShapeUpdate(G_{shape}, θ_i);

end for

6: **end for**

7: **return** $G_{shape} = [\theta_1, \theta_2, ..., \theta_m]$

---

4 References

Alici, G., Canty, T., Mutlu, R., Hu, W., and Sencadas, V. (2017). Modeling and experimental evaluation of bending behavior of soft pneumatic actuators made of discrete actuation chambers. Soft Robot. 5, 24–35. doi: 10.1089/soro.2016.0052

Natividad, R., Del Rosario, M., Chen, P. C. Y., and Yeow, C. H. (2018). A reconfigurable pneumatic bending actuator with replaceable inflation modules. Soft Robot. 5, 304–317. doi: 10.1089/soro.2017.0064

Pauly, J., and Midha, A. (2006a). Pseudo-Rigid-Body Model Chain Algorithm: Part 1 — Introduction and Concept Development, 173–181. doi: 10.1115/DETC2006-99460
